# Allotropy in ultra high strength materials

**DOI:** 10.1038/s41467-022-30845-z

**Published:** 2022-06-09

**Authors:** A. S. L. Subrahmanyam Pattamatta, David J. Srolovitz

**Affiliations:** 1 Department of Mechanical Engineering, The University of Hong Kong, Hong Kong SAR, China; 2 Greater Bay Joint Division, Shenyang National Laboratory for Materials Science, Hong Kong SAR, China; 3 International Digital Economy Academy (IDEA), Shenzhen, China

**Keywords:** Computational methods, Metals and alloys, Statistical mechanics, Method development

## Abstract

Allotropic phase transformations may be driven by the application of stresses in many materials; this has been especially well-documented for pressure driven transformations. Recent advances in strengthening materials allow for the application of very large shear stresses as well – opening up vast new regions of stress space. This means that the stress space is six-dimensional (rather than one for pressure) and that phase transformations depend upon crystal/grain orientation. We propose a novel approach for predicting the role of the entire stress tensor on phase transformations in grains of all orientations in any material. This multiscale approach is density functional theory based and guided by nonlinear elasticity. We focus on stress tensor dependent allotropic phase transformations in iron at high pressure and ultra-fine grained nickel and titanium. The results are quantitatively consistent with a range of experimental observations in these disparate systems. This approach enables the balanced design of high strength-high ductility materials.

## Abstract (web summary)

Here the authors propose a crystal thermodynamics framework describing the tensor stress induced phase transformations in solids based on nonlinear elasticity and first principles calculations. The proposed approach enables balanced design of high-strength, high-ductility materials.

## Introduction

Stronger and tougher materials are key to many advances in modern technology. While multiple routes are available to strengthen materials^1^, simultaneously achieving both high strength and toughness remains elusive. Strength refers to the ability of the material to resist plastic deformation (irreversible shape change) and toughness is the ability to absorb energy and plastically deform without fracturing. While the classical theoretical limit on material strength (yield stress) is roughly a tenth of the shear modulus^2^, commonly achievable strengths are only a small fraction of this. In addition to plastic deformation and fracture, stress can also be relieved by phase transformations (PT). PTs can therefore be exploited to toughen a material (e.g., transformation toughening^3^ and transformation-induced plasticity^4^).

Pressure-induced PTs have been studied extensively over the past half-century (e.g., in diamond anvil cells) to investigate common and exotic phases (e.g., in geological and extra-terrestrial materials). The focus on hydrostatic pressure (*p*), is associated, partly with geological conditions, and the experimental difficulties of generating shear stress as large as achievable hydrostatic stresses^5,6^. For metals, this is because yield strengths are comparatively small, i.e., dislocations (plastic deformation carrying defects) move easily. Unlike in conventional materials, nanoscale materials (e.g., nanograined/nanotwinned materials^7,8^) are often dislocation-starved^9,10^ leading to strengths approaching theoretical limits^11,12^; these are ultra-high-strength materials. The ability of ultra-high-strength materials to support large non-hydrostatic (shear) stresses, provides a means of accessing stress-induced PTs, unachievable in conventional (low strength) materials. Thus stress-induced PTs provide novel routes to overcoming the classical strength-toughness trade-off^13^ in a wide class of materials.

The effect of shear stresses on PTs is not limited to metals. Indeed, shear-induced PTs (transformation toughening) in zirconia are now well known (e.g., see refs. ^14–17^); the PT from tetragonal to monoclinic phases in MgO-partially stabilized zirconia (Mg-PSZ) and CeO_2_-stabilized tetragonal zirconia polycrystals (Ce-TZP) can be triggered by non-hydrostatic stresses and the transformation pressure is sensitive to shear. This effect has also received theoretical attention; e.g., several phenomenological models were proposed^18,19^ to describe the tetragonal to monoclinic PTs in zirconia under combined hydrostatic tension and shear.

While diamond anvil cell PT experiments focus largely on hydrostatic stress effects (shear stresses are limited by pressure media, gaskets, or sample strength), PT pressure measurements exhibit large scatter, frequently associated with shear stresses^5,6^. Rotational diamond anvil cell experiments^20^ confirm that shear alters transformation pressures^21^. For example, while iron undergoes a *p*-induced ferromagnetic body-centered cubic (bcc) to nonmagnetic hexagonal close-packed (hcp) PT, experimental studies^22–24^ yield widely differing transformation pressures (ranging from 8.6 to 15.3 GPa^25–28^); the non-hydrostaticity originates from finite strength of pressure media. Similar observations were reported for other materials (e.g., Zr and BN^29–31^).

Compelling evidence for shear-induced PTs also comes from recent experiments on nanocrystalline nickel^32^. Face-centered cubic (fcc) Ni is exceptionally stable; no PTs were observed in bulk specimens for *p* ≤ 200 GPa^33^ (hcp Ni was seen in very thin hetero-epitaxial Ni films^34,35^). Luo et al.^32^ observed that 5–10% of the grains in polycrystalline Ni (grain size below 17 nm) were entirely hcp while surrounding grains were fcc. The ultra-high-strength of such nanograined Ni admits extremely large non-hydrostatic stresses and the sensitivity of the PT to grain orientation demonstrates that the PT is sensitive to both shear stress and crystal orientation. Another example of stress-induced PTs is observed in titanium (hcp → fcc) in high energy mechanical attrition in ball mill^36^, cryogenic plane strain compression^37^, rolling polycrystal specimens^38^, and molecular dynamics simulations of nano-pillars under tension^39^. The PT mechanism is still debated although some mechanisms were proposed based on DFT studies^40^.

In this article, we propose a general framework to understand and predict the effect of the entire (tensor) stress state on PTs; large non-hydrostatic stresses are accessible only in ultra-high-strength materials. Our approach is based on (nonlinear) elasticity and density functional theory (DFT). The results shed light on novel pathways to PTs. We seek the lower bound on the stresses required for thermodynamic stabilization of a new phase and apply it to PTs in polycrystals.

Consider the diffusionless PTs from a parent phase *α* to a daughter phase *β* driven by Gibbs free energy reduction. The Gibbs free energy (hyper-) surface may have multiple minima corresponding to different stable/metastable crystal structures. As the stress (or temperature) varies, the Gibbs free energy surface morphs; new minima may appear, and existing minima may deepen/recede leading to a switch in the global minimum^41^. While changes in minima depths may lead to first-order PTs, the disappearance of a minimum may yield higher-order PTs.

We focus on isothermal, stress-induced PT thermodynamics in bulk phases (i.e., neglect interface energies and barriers between metastable states). In *p*-driven transformations, the PT occurs at a critical, scalar stress (pressure). However, PTs in general depend on all six stress components (the stress tensor is symmetric) such that the critical stress is a hyper-surface. We consider all possible transformations from each energy minimum in the parent phase to energy minima in daughter phases consistent with a particular transformation mechanism.

The mechanical response of a crystal subjected to a particular (non-hydrostatic) loading is anisotropic and depends on its orientation. A crystal in the phase *α* under an applied stress may transform to another phase *β* (that respects a transformation mechanism) if the Gibbs’s free energy of *β* is lower than that of *α*. In such a case the PT is said to be thermodynamically feasible. In the current work, we limit our discussion to zero temperature. Thus the Gibbs free energy reduces to the enthalpy *H*. Nevertheless our formulation is quite general and can be extended to include finite temperature contributions to the free energy. This is typically done through the quasi-harmonic approximation in DFT-based calculations. Thus stress-induced PTs in single crystals can be formulated as a problem of minimization of *H*. We consider the parent phase *α* in the stress-free state as the reference configuration. We also assume that the applied stress produces a homogeneous deformation inside the crystal and the motion of the elastic body is described by the total deformation gradient which can be multiplicatively decomposed as,

1 $${{{{{{{\boldsymbol{F}}}}}}}}={{{{{{{{\boldsymbol{F}}}}}}}}}_{e}{{{{{{{{\boldsymbol{F}}}}}}}}}_{t}$$

where the ***F***_*e*_ is the elastic component and ***F***_*t*_ is the non-elastic stress-free transformation component of the total deformation gradient. ***F***_*t*_ is a mapping the takes the reference state to the daughter phase in its stress-free configuration for a certain orientation relation. In the absence of a PT, the deformation is completely elastic i.e., ***F***_*t*_ = ***I***, where ***I*** is the identity tensor.

The elastic strain in the crystal is given by the Green-Lagrange strain tensor ${{{{{{{{\boldsymbol{E}}}}}}}}}_{e}=\frac{1}{2}({{{{{{{{\boldsymbol{F}}}}}}}}}_{e}^{T}{{{{{{{{\boldsymbol{F}}}}}}}}}_{e}-{{{{{{{\boldsymbol{I}}}}}}}})$ (The superscript *T* indicates transpose). The enthalpy of the crystal per unit volume in the reference configuration is given by^42,43^,

2 $$h({{{{{{{\boldsymbol{P}}}}}}}},{{{{{{{{\boldsymbol{F}}}}}}}}}_{t})=\psi ({{{{{{{{\boldsymbol{E}}}}}}}}}_{e})-{{{{{{{\boldsymbol{P}}}}}}}}:\left({{{{{{{\boldsymbol{F}}}}}}}}-{{{{{{{\boldsymbol{I}}}}}}}}\right)$$

The first term *ψ*(***E***_*e*_) on the right-hand side is the total internal energy density which comprises the chemical and elastic contributions. It is dependent only on the elastic component of the strain and the transformation deformation gradient ***F***_*t*_ (if any) does not contribute to it. The second term represents the external work per unit reference volume expended in moving the boundaries of the system and bringing it into the current state of existence against external loads maintained at the current level. It is thus dependent on the total deformation gradient ***F*** consisting of the elastic (***F***_*e*_) and transformation (***F***_*t*_) components. ***P*** is the first Piola-Kirchoff (PK) stress tensor in the reference configuration (See Supplementary Notes for remarks on the enthalpy equation).

The thermodynamic restrictions yield the constitutive stress response ${{{{{{{\boldsymbol{P}}}}}}}}={{{{{{{{\boldsymbol{F}}}}}}}}}_{e}\frac{\partial \psi }{\partial {{{{{{{{\boldsymbol{E}}}}}}}}}_{e}}{{{{{{{{\boldsymbol{F}}}}}}}}}_{t}^{-T}$. Note that ${{{{{{{\boldsymbol{P}}}}}}}}{{{{{{{{\boldsymbol{F}}}}}}}}}_{t}^{T}={{{{{{{{\boldsymbol{F}}}}}}}}}_{e}\frac{\partial \psi }{\partial {{{{{{{{\boldsymbol{E}}}}}}}}}_{e}}$ is in-fact the first PK stress defined in the intermediate stress-free transformed configuration. So in principle for a prescribed ***P***, the constitutive relation (as a black-box either DFT, molecular statics, or elastic tensors) and the transformation deformation gradient ***F***_*t*_; one can numerically determine *h*. Instead of prescribing ***P*** we can also prescribe the Cauchy or true stress ***σ*** (symmetric) in the deformed configuration as done in the current work. The Cauchy stress is related to the first PK stress in the reference configuration through ${{{{{{{\boldsymbol{\sigma }}}}}}}}={J}^{-1}{{{{{{{\boldsymbol{P}}}}}}}}{{{{{{{{\boldsymbol{F}}}}}}}}}^{T}={J}^{-1}{{{{{{{\boldsymbol{P}}}}}}}}{{{{{{{{\boldsymbol{F}}}}}}}}}_{t}^{T}{{{{{{{{\boldsymbol{F}}}}}}}}}_{e}^{T}$ where $J=\det {{{{{{{\boldsymbol{F}}}}}}}}$. Thus computation of enthalpy for a given ***σ*** and ***F***_*t*_ (if any) can be carried out in a self-consistent manner: the strain with respect to the stress-free state, ***E***_*e*_ is iteratively corrected until the stress in the current configuration converges to ***σ***, yielding the elastic deformation ***F***_*e*_ and ***P*** (through ***σ*** and ***F***) (See Supplementary Methods). In DFT calculations we directly obtain *ψ*(***E***_*e*_) and in the case of (nonlinear) elasticity, knowledge of ***F***_*e*_ enables us to compute the elastic strain energy contribution to *ψ*(***E***_*e*_) with the chemical contribution in the stress-free state coming from DFT. The external work term in Eq. (2) can be computed from ***P*** and ***F***.

A PT from *α* to *β* is energetically feasible if it minimizes the enthalpy. As an example, we explicitly examine stress-induced PTs in Fe (bcc → hcp), Ni (fcc → hcp), and Ti (hcp → fcc). While the effect of pressure on PTs in Fe has been widely reported; the effect of shear on transformation pressures^25^ is unquantified. Although stress-induced PTs have been reported in Ti, Ni is a more extreme example since stress/*p*-induced PTs have not been reported; nonetheless recent experimental observations of hcp grains in ultra-high-strength, nanocrystalline Ni^32^ make this an interesting case. Under hydrostatic compression, the enthalpy of hcp Ni is always higher than fcc Ni, while hcp Fe has lower enthalpy than bcc Fe for *p* > 9.58 GPa^44^ (See Supplementary Figs. 1, 2 and 3). Although we focus on these three examples, our approach is general and is applicable to all materials that undergo displacive PTs. We consider the enthalpy density *h* of *α* and *β* phases in the full six-dimensional stress-space; and provide a simple procedure to identify thermodynamic PTs for all stresses and all crystal orientations of the parent phase *α*. Given the high dimensionality of the problem, we apply a nonlinear elasticity approach to guide quantitative DFT calculations.

We consider PTs between the *α* and *β* for a specific crystallographic orientation relationship (OR); as discussed below, relatively few ORs are experimentally observed between a pair of phases in diffusionless PTs. The common experimental observations of specific ORs implies that (i) such transformations are thermodynamically allowed and (ii) kinetically preferred (sufficiently low barriers).

The orientation of a crystal may be described as an ordered set of three rotations (e.g., Euler angles), an axis-angle pair, or an orientation matrix **g** ∈ *SO*(3). The orientation of *β* relative to *α* is represented by Δ**g**^*αβ*^. The *α* − *β* OR may also be specified in terms of a set of parallel planes (*hkl*)^*α*^ ∥ (*hkl*)^*β*^ and directions [*uvw*]^*α*^ ∥ [*uvw*]^*β*^ in the two crystal phases. Depending on the *α* and *β* crystal symmetries, multiple variants of *β* are possible for a given OR with *α*; hence it is a set of relative orientations {Δ**g**^*αβ*^} whose elements are related by *α* and *β* symmetries.

**Fig. 1:**
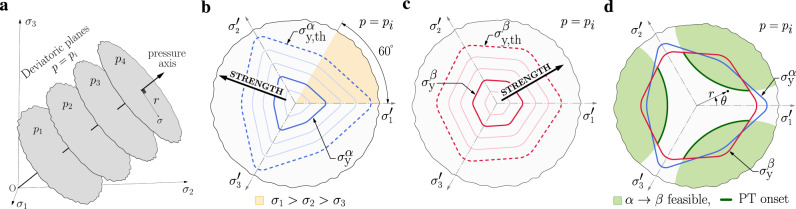
A schematic of the feasible phase transformation domains in stress-space. **a** Three dimensional principal stress-space represented by the *σ*_1_, *σ*_2_, and *σ*_3_ principal stress axes (*σ*_1_ = *σ*_2_ = *σ*_3_ is the *p*-axis, deviatoric planes are perpendicular to this axis). Panels **b**, **c** show envelopes outside of which *α* and *β* flow (yield) on a particular deviatoric plane. The axes ${\sigma }_{1}^{\prime},{\sigma }_{2}^{\prime}$ and ${\sigma }_{3}^{\prime}$ are projections of stress-space axes *σ*_1_, *σ*_2_, and *σ*_3_ onto the deviatoric plane. As the material is strengthened, the yield envelopes, ${\sigma }_{{{{{{{{\rm{y}}}}}}}}}^{\alpha }$ and ${\sigma }_{{{{{{{{\rm{y}}}}}}}}}^{\beta }$, expand towards the theoretical strength limits ${\sigma }_{{{{{{{{\rm{y}}}}}}}},{{{{{{{\rm{th}}}}}}}}}^{\alpha }$ and ${\sigma }_{{{{{{{{\rm{y}}}}}}}},{{{{{{{\rm{th}}}}}}}}}^{\beta }$. **d** Stress-space regions for which *α* → *β* PTs are feasible (shaded light green). The PT onset boundary is shown in dark green.

The first task is to be able to evaluate *h* of an oriented crystal as a function of stress. We express stress in principal stress-space (i.e., Haigh-Westergaard space), where any stress is expressed as a principle stress triplet *σ* = (*σ*_1_, *σ*_2_, *σ*_3_) (see Fig. 1a) or as *σ* = (*r*, *θ*, *p*) (see Fig. 1d). The same basis vectors of the stress-space are taken to express the orientations of the crystals. The enthalpy density *h*^*α*^(***σ***) of *α* grain with orientation **g**_*i*_ may be evaluated using any method: elasticity, molecular mechanics, DFT, .... in conjunction with Eq. (2). Note that the state of stress ***σ*** in grain is dependent on the stress triplet *σ* in the stress-space and the grain orientation **g**. Thus ***σ*** = ***σ***(*σ*, ***g***) is obtained through a change of basis. The pressure ($p=-(1/3){{{{{{{\rm{Tr}}}}}}}}({{{{{{{\boldsymbol{\sigma }}}}}}}})$) axis corresponds to the direction *σ*_1_ = *σ*_2_ = *σ*_3_ in stress-space; perpendicular to this axis are deviatoric (shear stress) planes. Any stress state can be decomposed into two parts: a distance along the *p*-axis and a vector lying in the corresponding deviatoric plane (see Fig. 1a). The deviatoric stress magnitude is the length of this vector (von Mises stress is $\sqrt{3/2}$ its length).

Since plasticity in most metals is controlled by dislocation motion and such a motion is driven by shear, we may represent the onset of plastic deformation as a closed contour on the deviatoric stress plane (i.e., the intersection of the yield surface with the deviatoric plane); the contour shape depends on *p*. For elastic-ideally plastic material, the crystal will be purely elastic inside this contour, and stresses outside it are unrealizable. The *α* and *β* yield strength contours, ${\sigma }_{{{{{{{{\rm{y}}}}}}}}}^{\alpha }$ and ${\sigma }_{{{{{{{{\rm{y}}}}}}}}}^{\beta }$, are shown schematically in Fig. 1b, c. The contours have three-fold rotation symmetry and three mirrors; the irreducible deviatoric stress domain is highlighted in yellow (See refs. ^45,46^ for a general discussion of stress-space and its structure). As the strength of the material increases, the yield surfaces expand outwards toward their theoretical limits ${\sigma }_{{{{{{{{\rm{y}}}}}}}},{{{{{{{\rm{th}}}}}}}}}^{\alpha }$ and ${\sigma }_{{{{{{{{\rm{y}}}}}}}},{{{{{{{\rm{th}}}}}}}}}^{\beta }$. Note, that while metals commonly deform by dislocation motion, this is less common in ceramics. In ceramics, and some metals, twinning is also important and failure by fracture may precede the onset of these deformation mechanisms. Here, we only consider the effect of stress on PTs.

The next task is to identify “feasible” transformation domains in stress-space; i.e., the set of stresses for which an *α* grain can transform to a *β* variant. A transformation is “feasible” if at a point in stress-space there is at least one *α* grain for which a transformation to a suitably oriented *β* grain (respecting the transformation mechanism; OR) lowers *h*. Figure 1d shows feasible PT domains (light green) on the *p*_*i*_ deviatoric plane and the *α* and *β* yield envelopes. Since stress states outside the yield envelope are inaccessible, PTs outside these envelopes are unachievable. Hence, *α* → *β* PTs can only occur for stress states (light green) that lie within both yield envelopes. The PT onset is given by the inner boundary of the feasible transformation domain (highlighted in dark green). Of particular interest are points on this boundary closest to the *p*-axis; these stress states are the minimum deviatoric PT stresses at *p*_*i*_.

We consider PTs within a polycrystal in which each grain is described in terms of its crystallographic orientation; in this sense, a polycrystal is a distribution of grain orientations. Firstly, in principle, we can evaluate *h*^*α*^ for all possible grain orientations **g** ∈ *SO*(3) and stresses *σ* in the stress-space. For each *α* grain with an orientation **g**, we can find relative orientations and transformations of all possible daughter variants obtained through a PT mechanism (i.e., OR). Thus, in principle, we can also evaluate the enthalpies *h*^*β*^ of all daughter variants obtained from each parent grain.

**Fig. 2:**
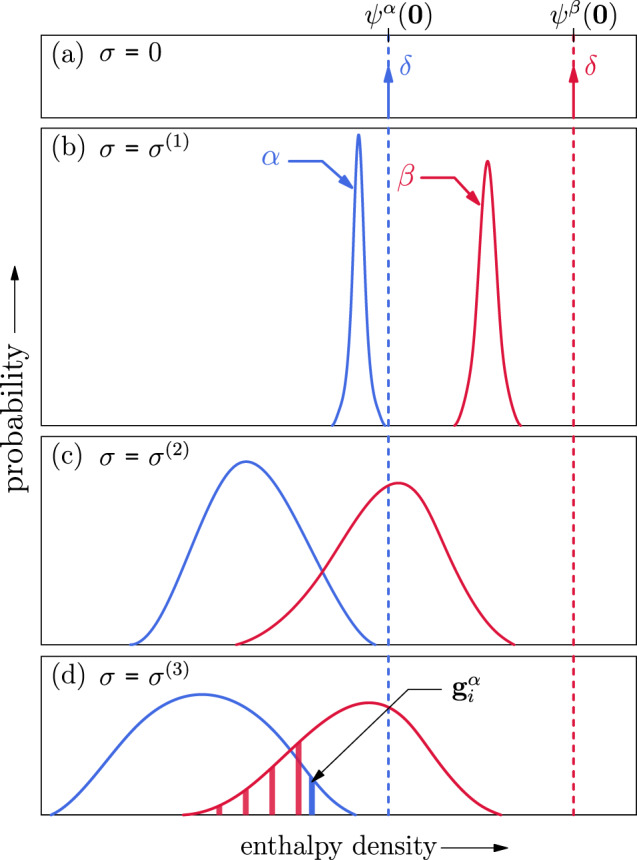
A schematic of stress dependence of the enthalpy density distribution. **a** At zero stress, all grain orientations have the same enthalpy density equal to the chemical binding energy density of the stress-free state *ψ*(***E***^*e*^ = ***0***). **b**–**d** Enthalpy density distributions for increasing stress. **d** Transformation from a parent grain (blue bar) with orientation ${{{{{{{{\boldsymbol{g}}}}}}}}}_{i}^{\alpha }$ to variants in daughter phase orientations restricted by an OR (red bars).

In the stress-free state, all grains of a phase have the same enthalpy density *h* = *ψ*(***E***^*e*^ = ***0***). This is shown by Dirac-delta functions in Fig. 2a. When a stress is applied, say to all grains in *α* phase, since *h* is a function of grain orientation (all crystals are anisotropic); this implies that the distribution of *h* for a polycrystal broadens and also shifts to the left as shown by the blue curve in Fig. 2b.

For understanding, we also schematically show the enthalpies of all daughter grains *β* obtained through an OR from each of the parent grains. This distribution in shown by the red curve in Fig. 2b. But in contrast to the parent blue curve, a pronounced feature of the daughter curve is that it shifted to the left. This is due to the additional contribution to the external potential term due to the transformation strain apart from elastic straining (see the second term in Eq. (2)). Monotonically increasing the applied stress further broadens the distributions and shifts them to the left (cf. Fig. 2b–d). It may also lead to overlap between the distributions (see Fig. 2c). PTs are more likely when more overlap occurs (Fig. 2d).

In Fig. 2b, the enthalpy of each *β* grain is larger than that of any *α* grain (no *α* → *β* PT). Increasing stress increases the overlap between the *h* distributions (Fig. 2c, d), implying that some *β* variants corresponding to an *α* may have lower enthalpies at this stress. For a specific OR between *α* and *β* phases, a parent grain ${{{{{{{{\bf{g}}}}}}}}}_{i}^{\alpha }$ (represented by the vertical blue bar in Fig. 2d) may only transform to the subset of grains *β* that are consistent with the OR (the dark red vertical bars in Fig. 2d). At any stress *σ* the fraction of *α* grains that may transform to *β* is obtained by evaluating the possibility of PT for each parent grain. For fcc → hcp PTs, the most commonly observed ORs are basal and prismatic and for bcc → hcp PTs the most widely observed OR is associated with the Burgers mechanism (as summarized in Table 1). The corresponding deformation gradients^47^***F***_*t*_ describing the PT are given in Supplementary Methods. The approach to PTs described here is applicable to any material and any OR (including twinning with no phase change).

**Table 1:** Basal and prismatic orientation relations for fcc–hcp and the Burgers orientation relation for bcc–hcp PTs.

Type	Orientation relation	
Basal	${\left\{111\right\}}_{{{{{{{{\rm{fcc}}}}}}}}}\parallel {\left\{0001\right\}}_{{{{{{{{\rm{hcp}}}}}}}}}$	${\left\langle 1\overline{1}0\right\rangle }_{{{{{{{{\rm{fcc}}}}}}}}}\parallel {\left\langle 11\overline{2}0\right\rangle }_{{{{{{{{\rm{hcp}}}}}}}}}$
Prismatic	${\left\{1\overline{1}0\right\}}_{{{{{{{{\rm{fcc}}}}}}}}}\parallel {\left\{10\overline{1}0\right\}}_{{{{{{{{\rm{hcp}}}}}}}}}$	${\left\langle 001\right\rangle }_{{{{{{{{\rm{fcc}}}}}}}}}\parallel {\left\langle 0001\right\rangle }_{{{{{{{{\rm{hcp}}}}}}}}}$
Burgers	${\left\{110\right\}}_{{{{{{{{\rm{bcc}}}}}}}}}\parallel {\left\{0001\right\}}_{{{{{{{{\rm{hcp}}}}}}}}}$	${\left\langle \overline{1}11\right\rangle }_{{{{{{{{\rm{bcc}}}}}}}}}\parallel {\left\langle \overline{2}110\right\rangle }_{{{{{{{{\rm{hcp}}}}}}}}}$

## Results

We now apply this method to understand the effects of applied stress on PTs in iron, nickel, and titanium. In each case, we pose the following questions: For which stress states ***σ*** does the PT occur? What is the smallest deviatoric stress that can initiate this PT? What is the effect of deviatoric stresses on PT as a function of *p*? What fraction of the grains transform at a point *σ* in the stress-space?

**Fig. 3:**
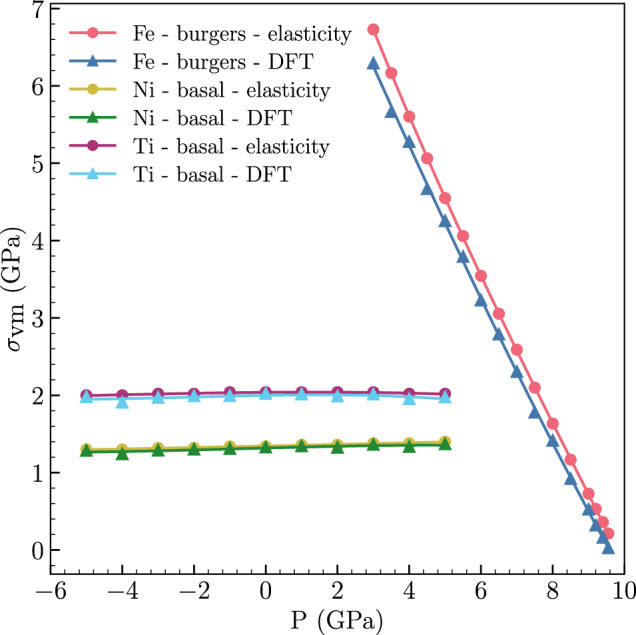
The minimum von Mises stress for the onset of phase transformation. The points on the PT onset boundaries closest to the *p*-axis for various pressures are computed for PTs in Fe, Ni and Ti using nonlinear elasticity and DFT.

Figure 3 presents the smallest shear (von Mises) stress needed to induce a PT in each metal as a function of pressure. The von Mises stress required to induce a PT in Fe decreases almost linearly with increasing pressure and goes to zero *p* = 9.58 GPa (the isentalphic pressure of bcc and hcp phases, see Supplementary Fig. 1). From our data the relationship between the pressure and minimum *σ*_vm_ in Fe is given by *p*_PT_ = 9.56 − 1.05*σ*_vm_ GPa. Thus the transformation pressure decreases with increasing shear; i.e., the trend observed in experiments^25,26^. Figure 3 demonstrates that the nonlinear elasticity estimates (red) of the onset of the PT in Fe is in close agreement with the more accurate DFT calculations. The nonlinear elasticity predictions slightly overestimate the true PT stresses; the deviation decreases with decreasing shear stress. This is consistent with the finite order of the nonlinear elasticity calculations.

The smallest shear stress required to induce a fcc → hcp PT in Ni (Fig. 3) shows that (i) no PT is observed in the absence of deviatoric stress at any pressure (examined), and (ii) the shear stress required to induce PT is very insensitive to pressure. These observations are associated with the fact that both the parent fcc and daughter hcp phases in Ni are close-packed and have a very small difference in volume per atom, unlike the bcc → hcp PT in Fe. In Ni, the transformation is shear-dominated (basal mechanism). Here we consider only basal mechanism fcc – hcp PTs. The fcc → hcp PT generally occurs at much lower shear stress in Ni, than the corresponding bcc → hcp PT in Fe (except at very high compression). This implies that nonlinear elastic prediction of the transformation stresses represents a better approximation (of the DFT results) in Ni than in Fe. Nonetheless, the nonlinear elastic predictions again overestimate the true (DFT) results.

The transformation stress trends for hcp → fcc in Ti are similar to those for fcc → hcp PT Ni, Fig. 3. This is because, like Ni, the two phases of Ti have nearly the same volume per atom. However, the shear stress required for the hcp → fcc PT in Ti is more than 30% larger than for the fcc → hcp PT in Ni. Normalized by the appropriate shear modulus, this difference is even larger. This can be understood by considering the large enthalpy difference at zero pressure between fcc and hcp phases in Ti ( ≈ 0.058 eV/atom) as compared to the zero pressure enthalpy difference between hcp and fcc phases in Ni ( ≈ 0.024 eV/atom).

**Fig. 4:**
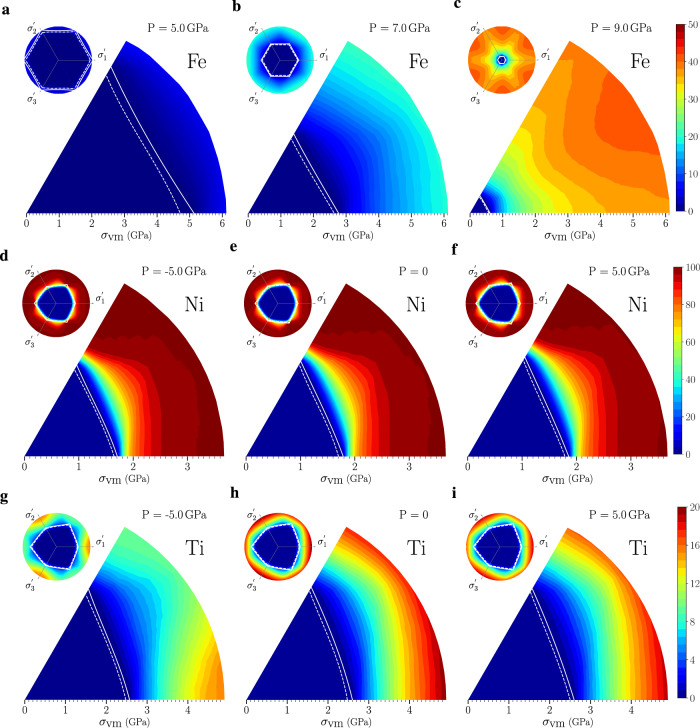
Percentage of transformed grains under stress. The polycrystal is un-textured and the phase transformation is computed from nonlinear elasticity using Sachs model. Panels **a**–**c** for bcc → hcp PT in Fe, **d**–**f** for fcc → hcp PT in Ni, and **g**–**i** for hcp → fcc PT in Ti.

Figure 4 shows the percentage of parent grains that will transform to the daughter phase on the deviatoric plane at several pressures for Fe (bcc → hcp), Ni (fcc → hcp), and Ti (hcp → fcc) based upon the nonlinear elasticity calculations. These data are based upon a Sachs model prediction (i.e., assumes all grains are at the same stress) and hence represent a lower bound on the transformation stresses. As seen in Fig. 3, the Ti and Ni results are nearly pressure-independent, while the fraction transformed is strongly pressure-dependent in Fe where the bcc and hcp phases have very different densities. No data are shown for Fe for *p* < 3.5 GPa since there is no bcc → hcp transformation. The dashed and solid white lines represent the stress for the onset of transformations based upon the DFT and nonlinear elastic predictions. These lines are close together, indicating again that the nonlinear elastic solution is in excellent agreement with the DFT predictions. These plots for all materials (including Fe) exhibit the three-fold symmetry of stress-space.

The recent experimental observations of Luo et al.^32^ in polycrystalline Ni with a gradient in grain size down to a few nanometers (formed by cryogenic surface mechanical grinding), showed that grains larger than 17 nm were fcc but some grains smaller than 17 nm were entirely hcp. The grain refinement during high-intensity cryogenic grinding likely occurs through extensive plastic deformation, leaving the near-surface region with biaxial stresses of order of the yield stress. The hardness of 17 nm grain size regions was ~6 GPa; assuming the yield stress is ~1/3 the hardness, this suggests a yield strength of ~2 GPa. Our theoretical prediction for the onset of fcc → hcp transformations is ~1.6 GPa. Given the rough nature of the experiment-based yield strength estimate, this is in excellent agreement. Luo et al. further showed that regions with smaller grains had higher hardness/yield strength and observed that the fraction of grains that were hcp were larger—again consistent with Fig. 4d–f. Our calculations slightly overestimate the fraction of grains transformed, compared with the experiments. This may be attributed to the crude estimate of yield stress from the hardness measurements and our overestimation of the fraction of grains transformed based upon the Sachs assumption (uniform stress) and nonlinear elasticity.

## Discussion

While PTs in many materials are known to occur at large pressures, experimental observations of PTs driven by large shear stresses are rare. This is largely because the magnitude of physically realizable shear stresses is limited by the strength of the material. Advances in materials science have made ultra-high strengths increasingly achievable. This points to the increasing importance of shear-driven PTs. Such PTs are currently being exploited to toughen modern alloys^3,4^. Shear-driven PTs are also widely observed at relatively low stress, e.g., in shape memory alloys and multiferroic oxides^48^, giving rise to large strains prior to failure.

A particularly interesting case is Fe, which shows a pressure-induced bcc → hcp PT over a wide range of pressures (8.6 ≤ *p* ≤ 15.3 GPa)^25,26^. Thermodynamically, this PT should occur at a single pressure; our DFT calculations suggest this occurs at *p* = 9.58 GPa (also see ref. ^44^). Several researchers have suggested^25–28^ that this large pressure range may be associated with the presence of shear in the experiments. Our theoretical/computational results demonstrate that the critical pressure can be reduced by the application of shear stresses from the shear free value of *p* = 9.58 GPa. The smallest transformation pressure observed in experiments was 8.6 GPa^26^. Based on our calculations, the shear stress required to decrease the transformation from 9.58 to 8.6 GPa is ~1 GPa. Given that the theoretical strength of Fe is ~7.5 GPa^49^, a yield stress of 1 GPa is readily achievable in some grains in nanocrystalline samples. For a single crystal, however, the shear stress must be applied in a favorable direction relative to the crystal orientation; applying the shear in the “wrong” sense could raise the transformation pressure.

The recent experiments on cryogenic surface mechanical grinding of Ni (grain sizes as small as 2 nm) showed the existence of grains that were entirely hcp while neighboring grains remained fcc^32^. This is surprising since bulk experiments show no *p*-induced fcc → hcp PT up to 200 GPa^33^ (theoretical calculations suggest none below 51 TPa^50^). However, small grains are ultra-high strong because of classic grain size strengthening and dislocation starvation. In a microstructure, where the mean grain size is less than 10 nm, only 5–10% of the grains were observed to be hcp with an increasing fraction with decreasing grain size. The hcp grains did not transform back to fcc upon annealing (1 h at 773 K) suggesting that the hcp crystal structure is thermodynamically preferred (as argued here) or, at least, deeply metastable.

Since the volume per atom in the hcp phase of is only 0.3% larger than fcc Ni under stress-free conditions, it is not surprising that no fcc → hcp PTs occur at high hydrostatic pressure in the absence of shear. The present results, however, suggest that Ni can transform from fcc → hcp under the application of shear stresses ~1.4 GPa at *p* = 0. Such a transformation can only occur for a limited range of stress states and for a fraction of the grains in the polycrystal (depending on grain orientation). This is consistent with the experimental observation that no more than 10% of the grains were hcp.

In summary, we proposed an approach for exploring the effect of the entire stress tensor on phase stability that is applicable to all materials systems. It is multiscale, in the sense, that it is DFT-based, but is guided by nonlinear elasticity calculations (parameterized by DFT). As the strength of materials continues to rise through innovations in microstructure and alloy development, the role of shear stresses in inducing PTs becomes increasingly significant; in some cases, more important than pressure. Our predictions are validated by comparison with experiments for different allotropic PTs in (1) Fe (for which there is copious data on *p*-induced PTs) and (2) Ni (where pressure has little effect on PTs) and (3) Ti (where some experimental evidence exists). In most practical situations, our approach can be applied through the application of nonlinear elasticity and DFT calculations are only required to determine higher-order elastic constants (extant experimental data are of variable quality). This means our approach is sufficiently efficient for application in material design and exploration, including the balancing of microstructure/alloying-based strengthening with PT-based approach for balancing high strength and high toughness.

## Methods

We present a multiscale methodology for predicting the stress conditions for which PTs can occur in polycrystalline materials. In particular, we describe the construction of the *α* → *β* PT boundary in stress-space (Fig. 1d). Since PTs may occur at large stress, accurate and reliable calculation of the PT conditions is only possible through first principles (DFT) calculations. Linear elasticity computations can provide useful guidance but are unreliable at large stresses/deformations since the elastic constants usually are fitted to small strains to reproduce the stress-strain response around the equilibrium configuration. Similarly, atomistic calculations are unreliable since interatomic potentials are routinely fitted to (near) equilibrium data. Compared with these methods, DFT calculations are computationally expensive. Identifying stress states for feasible PTs involves calculating *h* for all possible *α* grain orientations and all possible *β* orientations (related through an OR) at each point *σ* in the stress-space. Clearly, this is not computationally tractable using DFT.

Rather, our approach is to first determine the PT onset boundary using nonlinear elasticity calculations and use the result to guide the DFT calculations. We proceed as follows:

- Determine the ground state energies, second and third-order elastic constants of *α* and *β* using DFT (see Supplementary Figs. 4 to 9 and Supplementary Methods).
- For each point *σ* in stress-space, determine if at least one grain transforms as follows:
  1. For each parent grain orientation **g**^*α*^ determine ${h}_{{{{{{{{\rm{el}}}}}}}}}^{\alpha }({{{{{{{\boldsymbol{\sigma }}}}}}}})$ (subscript “el" indicates value obtained from nonlinear elasticity) and the corresponding ${h}_{{{{{{{{\rm{el}}}}}}}}}^{\beta }({{{{{{{\boldsymbol{\sigma }}}}}}}},{{{{{{{{\boldsymbol{F}}}}}}}}}_{t})$ of all possible *β* grain variants **g**^*β*^ within the OR.
  2. If the enthalpy density of a *β* grain variant is less than the parent *α* grain, the *α*  →  *β* PT is feasible.
  3. Label the stress point as feasible.
- The inner envelope of the feasibility region (union of all feasible stress points) is the elastic PT onset boundary.
- Determine the PT onset boundary with DFT (see below) for (nonlinear) elastically determined grain orientations—highest enthalpy density grain ${{{{{{{{\bf{g}}}}}}}}}_{{{{{{{{\rm{el}}}}}}}}}^{\alpha }$ and its lowest enthalpy density variant ${{{{{{{{\bf{g}}}}}}}}}_{{{{{{{{\rm{el}}}}}}}}}^{\beta }$ at each point on the elastic PT onset boundary.

Each point on the PT onset boundary in stress-space corresponds to specific grain orientations, ${{{{{{{{\bf{g}}}}}}}}}_{{{{{{{{\rm{el}}}}}}}}}^{\alpha }$ and ${{{{{{{{\bf{g}}}}}}}}}_{{{{{{{{\rm{el}}}}}}}}}^{\beta }$. While the nonlinear elastic determination of the *α* and *β* energies is not accurate at large stresses, the nonlinear elastic predictions of which grain orientations have the maximum or minimum energies are reliable. To verify this, we compare the energies of several randomly oriented bcc Fe grains via both third-order elasticity and DFT for several stress states. As shown in Supplementary Fig. 10 and described in Supplementary Methods, nonlinear elasticity provides an excellent ordering of the energies of differently oriented grains.

The reliability of the nonlinear elasticity orientation predictions allows us to focus on the DFT determination of the PT boundary as a function of stress-state as follows: (1) Assume grain orientations ${{{{{{{{\bf{g}}}}}}}}}_{{{{{{{{\rm{DFT}}}}}}}}}^{\alpha }={{{{{{{{\bf{g}}}}}}}}}_{{{{{{{{\rm{el}}}}}}}}}^{\alpha }$ and ${{{{{{{{\bf{g}}}}}}}}}_{{{{{{{{\rm{DFT}}}}}}}}}^{\beta }={{{{{{{{\bf{g}}}}}}}}}_{{{{{{{{\rm{el}}}}}}}}}^{\beta }$. (2) Describe a point on the deviatoric plane at pressure *p* by (*r*, *θ*) where *r* = *τ* is the deviatioric radius; calculate ${h}^{\alpha }({{{{{{{\boldsymbol{\sigma }}}}}}}}(\sigma (r,\theta ,p),{{{{{{{{\bf{g}}}}}}}}}_{{{{{{{{\rm{DFT}}}}}}}}}^{\alpha }))$ and ${h}^{\beta }({{{{{{{\boldsymbol{\sigma }}}}}}}}(\sigma (r,\theta ,p),{{{{{{{{\bf{g}}}}}}}}}_{{{{{{{{\rm{DFT}}}}}}}}}^{\beta }),{{{{{{{{\boldsymbol{F}}}}}}}}}_{t})$ of the oriented *α* and *β* grains. (3) If along some radius *r*(*θ*), *h*^*β*^(*r*) = *h*^*α*^(*r*) this is the PT onset (at *θ*). The locus of all such points corresponds to the PT onset boundary on each deviatoric plane. For some *θ*, no PT occurs, while in others, either the *α* or *β* phases may reach the DFT theoretical yield (crystal loses stability) prior to PT (in such a case, no PT will occur). Similarly for each phase, the locus of points corresponding to the loss of stability determines the (DFT) theoretical strength of the material for those grain orientations, ${{{{{{{{\bf{g}}}}}}}}}_{{{{{{{{\rm{DFT}}}}}}}}}^{\alpha }$ and ${{{{{{{{\bf{g}}}}}}}}}_{{{{{{{{\rm{DFT}}}}}}}}}^{\beta }$ (See Supplementary Methods for a detailed discussion on the identification of phase transformation (PT) onset boundaries).

All DFT calculations were performed using the plane-wave basis DFT code, VASP^51^ with projector augmented wave (PAW) potentials^52,53^ in the PBE generalized gradient approximation (GGA)^54^. We employ a plane-wave energy cutoff of 700 eV, electronic self-consistency convergence tolerance of 10^−8^ eV, and a force convergence tolerance of 10^−4^ eV/Å for structural relaxation for Ni, Ti, and Fe in phase transformation calculations. For the calculations involving Ti and Fe we incorporate 3*s*, 3*p*, 3*d*, and 4*s* as valence states, and for calculations involving Ni we incorporate 3*p*, 3*d*, and 4*s* as valence states. Stress control is achieved through iterative rescaling of the supercell size and shape until convergence to the applied stress is achieved.

## Supplementary information

Supplementary Information

## Data Availability

The authors declare that the main data supporting the findings of this study such as the lattice parameters, elastic constants, orientation relations, and transformation strains, are available within the paper and the supplementary information. Any additional data that support the findings of this study are available from the corresponding author upon request.
