# Supplementary material for: Allotropy in ultra high strength materials
Source: Nat Commun. 2022 Jun 9;13:3326. doi: 10.1038/s41467-022-30845-z (PMC9184473; doi:10.1038/s41467-022-30845-z)
Supplement: Supplementary file 1 — Supplementary Information [file 41467_2022_30845_MOESM1_ESM.pdf]

Supplementary Information

Allotropy in ultra high strength materials

A. S. L. Subrahmanyam Pattamatta et al.

Supplementary Figures

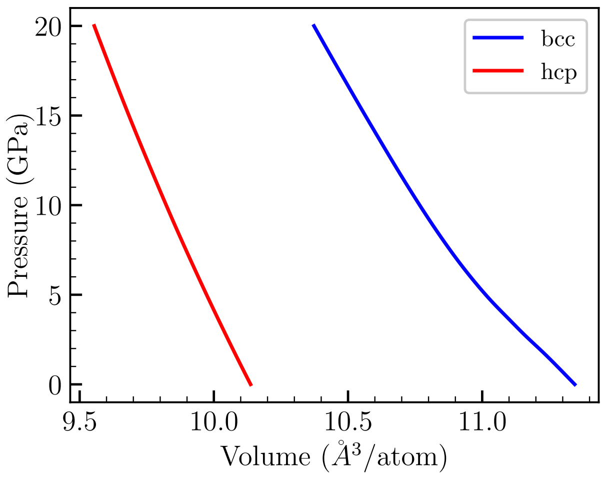

(a) Pressure versus volume of bcc and hcp phases.

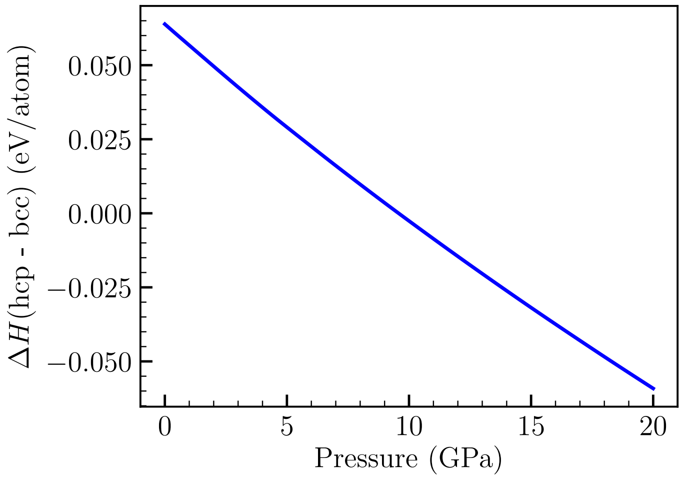

(b) Enthalpy difference between hcp and bcc phases.

Supplementary Figure 1: Pressure – volume and enthalpies of Iron. The body centered cubic (bcc) and hexagonal close packed (hcp) phases of Fe are subjected to various volumetric strains (relax lattice vectors and internal degrees of freedom while holding the volume fixed) and the internal energy Ψ and pressure p are computed using DFT. The enthalpy per atom is $H = \Psi + pV$, where Ψ is the internal energy per atom, V is the volume per atom at pressure p. Due to the difference in packing fractions of the phases, as evident from the plots, bcc phase of Fe has a larger volume compared to hcp phase at all pressures under consideration. The enthalpy difference indicates the relative thermodynamic stability under a hydrostatic loading. Both bcc and hcp phases of Fe have equal enthalpies at a pressure of 9.58 GPa. Above 9.58 GPa the hcp phase of Fe is more stable than the bcc phase.

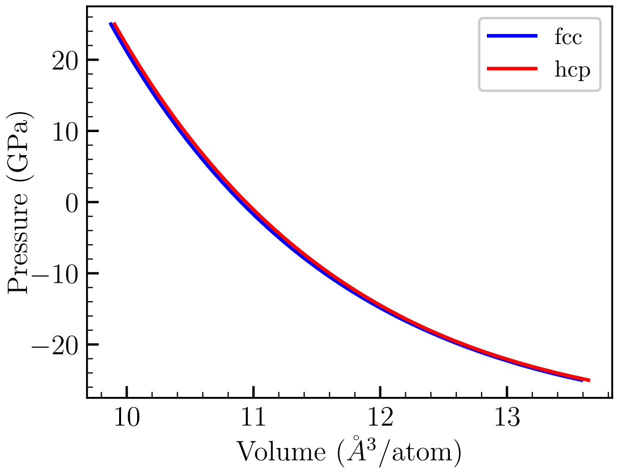

(a) Pressure versus volume of fcc and hcp phases.

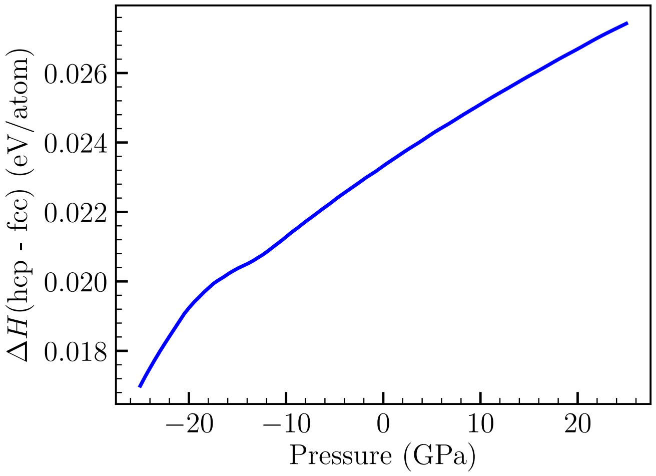

(b) Enthalpy difference between hcp and fcc phases.

Supplementary Figure 2: Pressure – volume and enthalpy differences of the face centered cubic (fcc) and hcp phases of Nickel. It can be observed that there is a very little difference in volume per atom between fcc and hcp phases.The enthalpy difference between hcp and fcc phases of Ni is always positive, indicating that the fcc phase of Ni is relatively stable compared to hcp phase at all pressures under consideration.

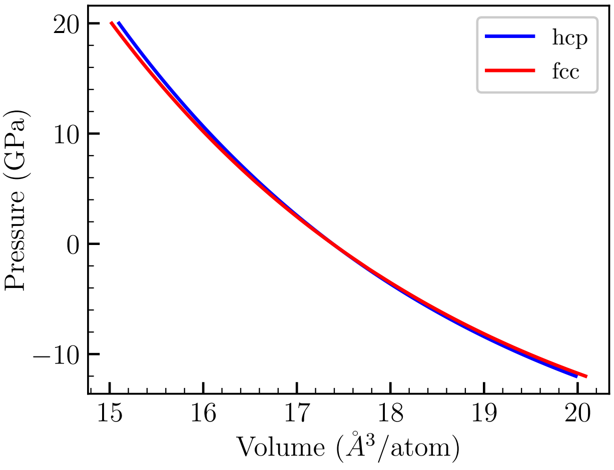

(a) Pressure versus volume of hcp and fcc phases.

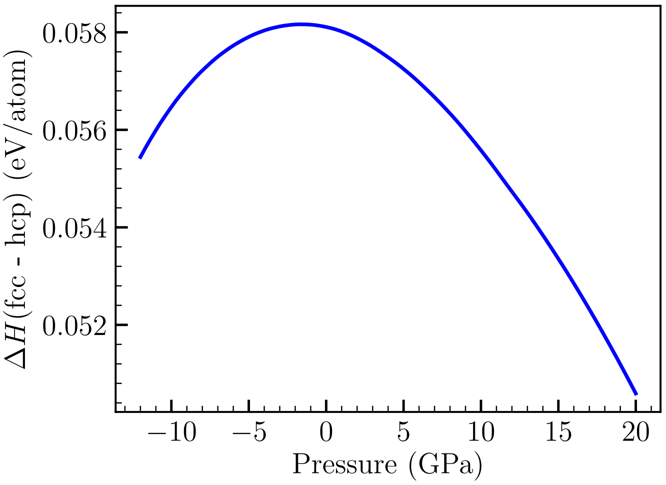

(b) Enthalpy difference between fcc and hcp phases.

Supplementary Figure 3: Pressure – volume and enthalpy differences of the hcp and fcc phases of Titanium.

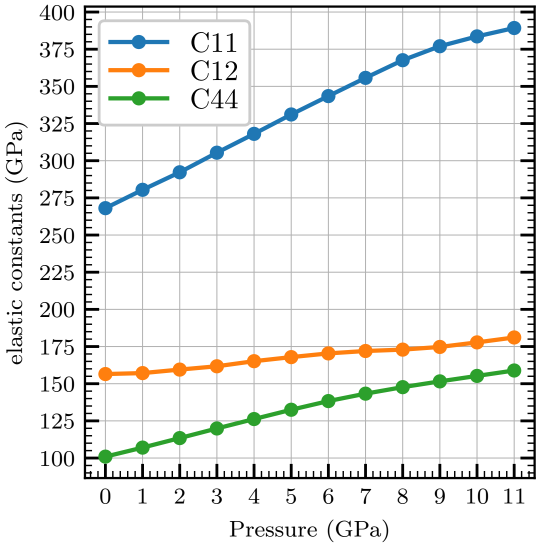

(a) Second order elastic constants

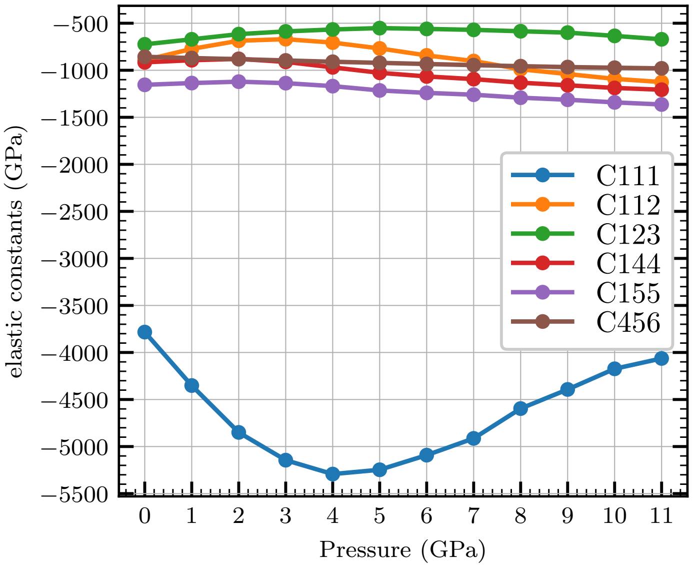

(b) Third order elastic constants

Supplementary Figure 4: Elastic constants of bcc Iron as a function of pressure.

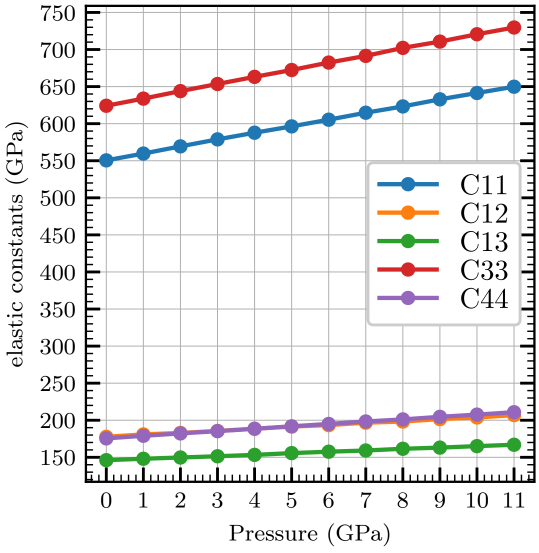

(a) Second order elastic constants

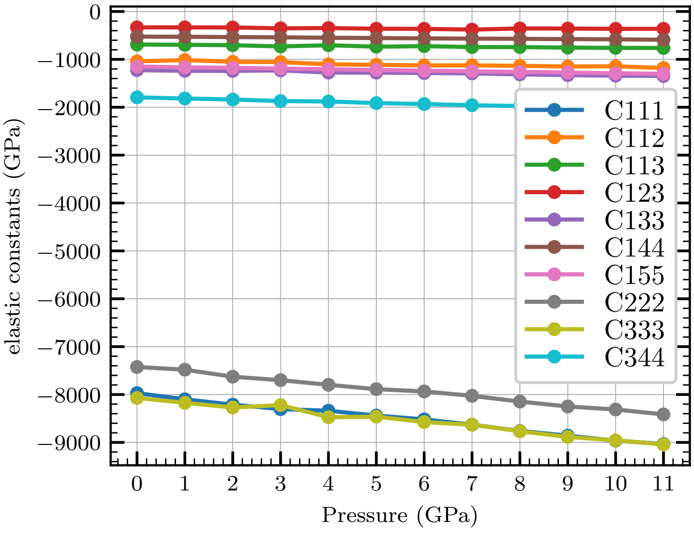

(b) Third order elastic constants

Supplementary Figure 5: Elastic constants of hcp Iron as a function of pressure.

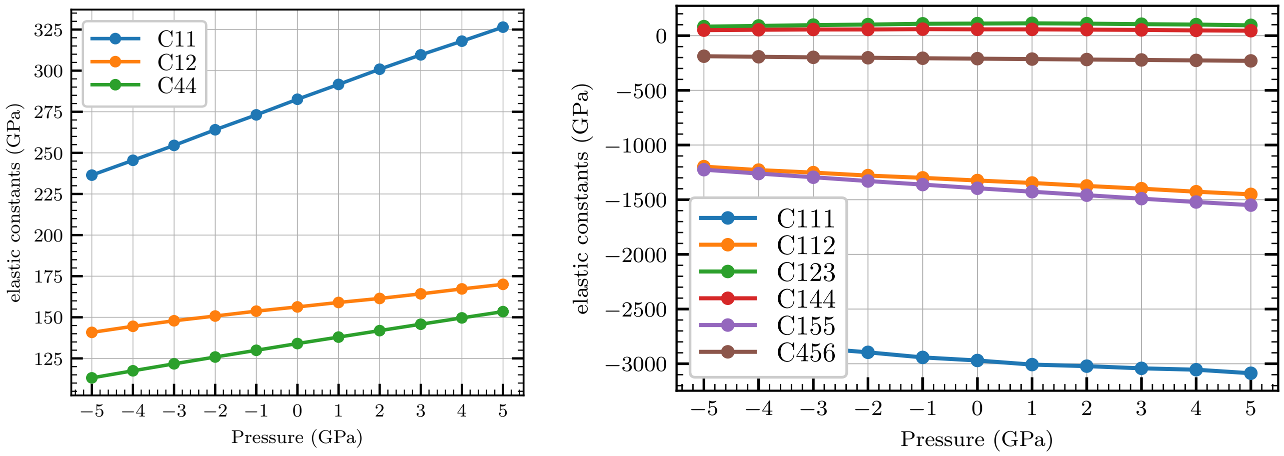

(a) Second order elastic constants

(b) Third order elastic constants

Supplementary Figure 6: Elastic constants of fcc Nickel as a function of pressure.

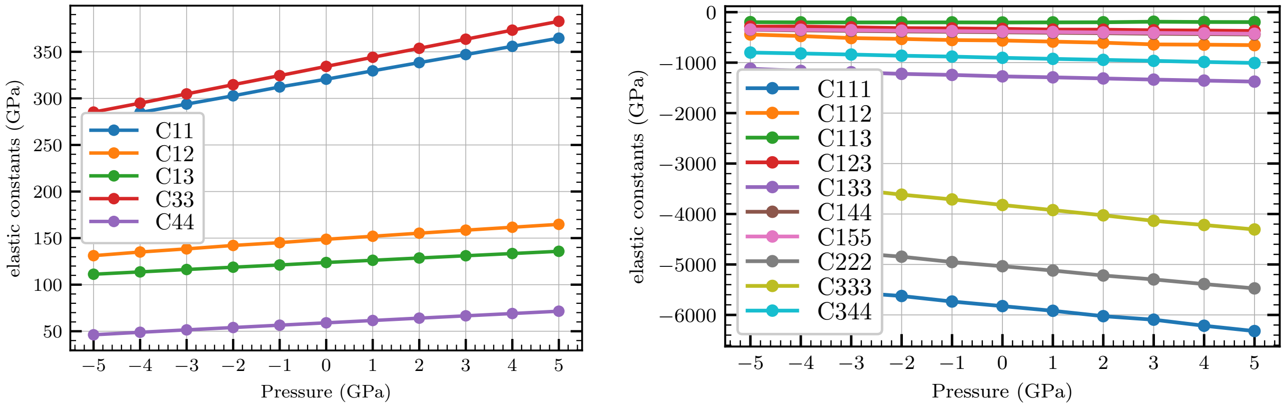

(a) Second order elastic constants

(b) Third order elastic constants

Supplementary Figure 7: Elastic constants of hcp Nickel as a function of pressure.

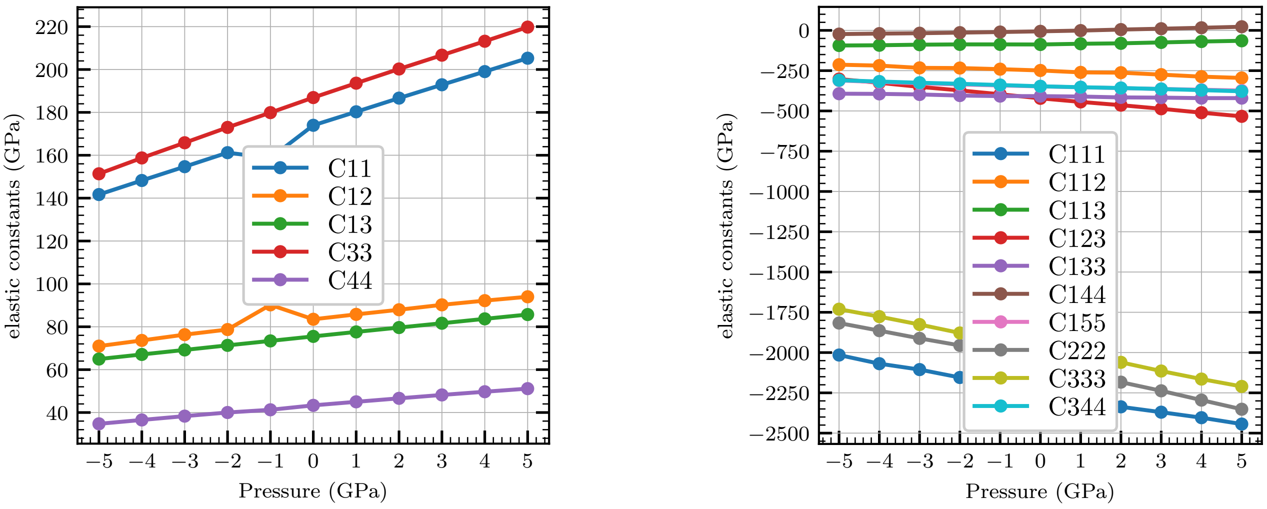

(a) Second order elastic constants

(b) Third order elastic constants

Supplementary Figure 8: Elastic constants of hcp Titanium as a function of pressure.

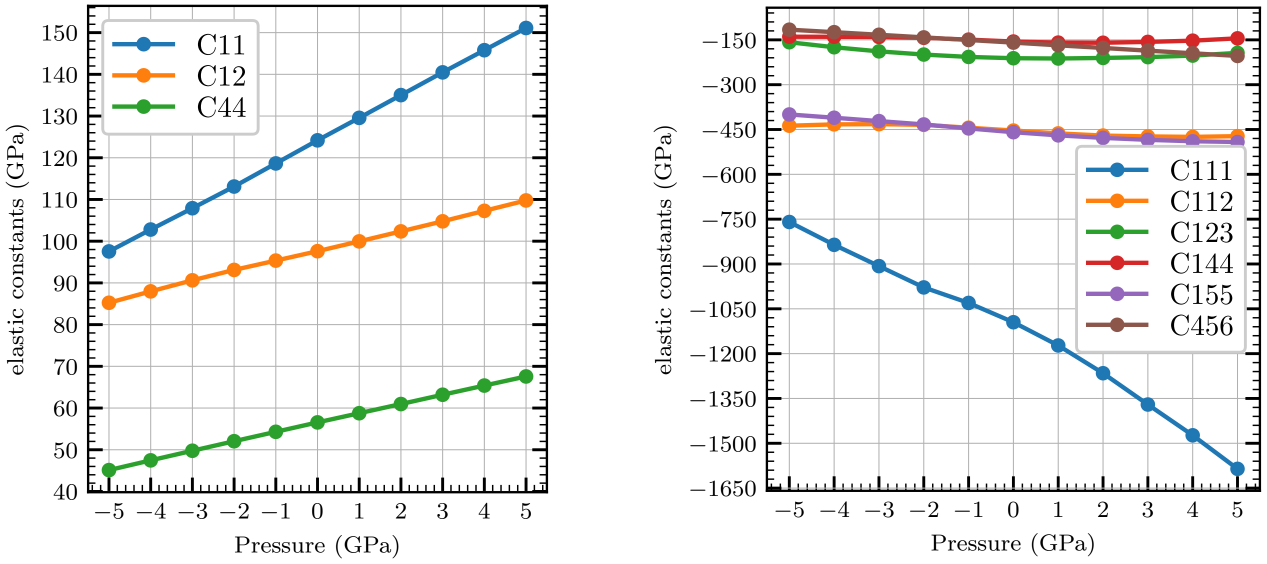

(a) Second order elastic constants

(b) Third order elastic constants

Supplementary Figure 9: Elastic constants of fcc Titanium as a function of pressure.

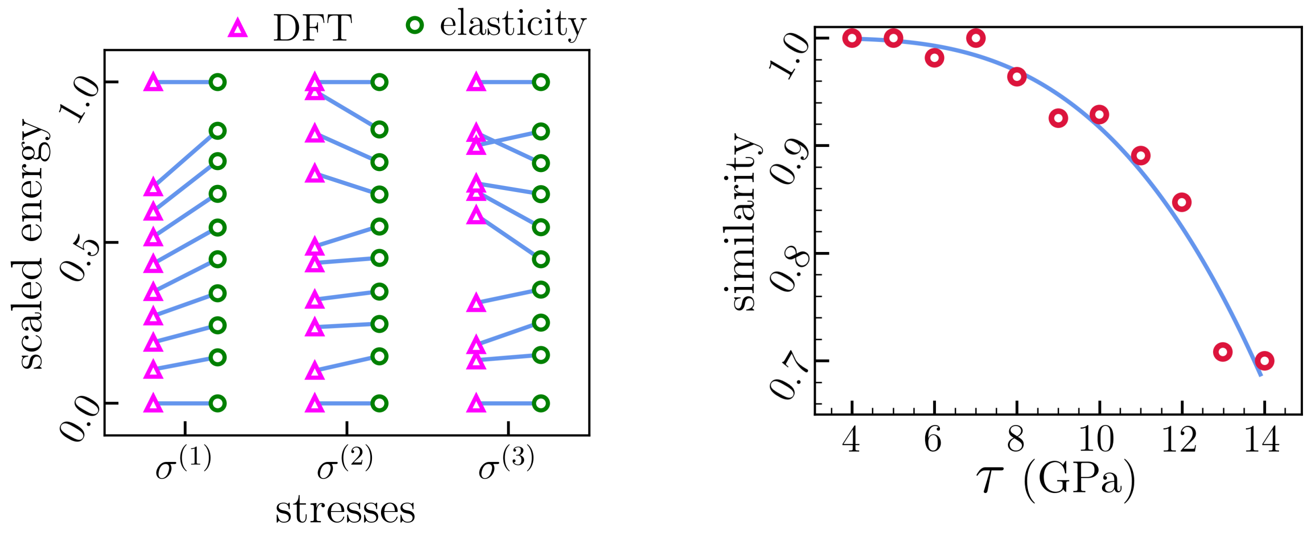

(a) **(b)**

Supplementary Figure 10: Levenshtein similarity. (a) Scaled energies from DFT and elasticity for several grain orientations. Comparison of internal energies predicted by DFT and elasticity in bcc iron at three randomly chosen stress states $\sigma^{(1)} = (3.33, -0.68, -2.43)$ GPa, $\sigma^{(2)} = (4.08, 4, 08, -8.16)$ GPa and $\sigma^{(3)} = (6.67, 1.87, -8.54)$ GPa at P=0. (b) Average Levenshtein similarity as a function of deviatoric radius τ. See Supplementary Methods for Levenshtein similarity.

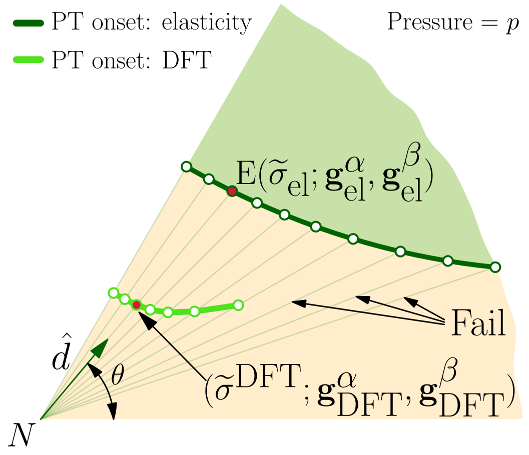

Supplementary Figure 11: A schematic of the radial continuation procedure. The continuation direction is indicated by the green arrow $\hat{d}$ making an angle θ. The deviatoric plane corresponds to a pressure p.

Supplementary Methods

Calculation of elastic constants of Iron, Nickel and Titanium

The elastic constant tensors are computed by applying various strain stencils with a large strain magnitude of 0.045 i.e. 4.5%. Also since the third order elastic constants are very sensitive to the k-spacing and kinetic energy cutoff in DFT, we used a very fine k-spacing density of $0.02 \times 2\pi$ per Å and an increased energy cutoff of 1000 eV for evaluation of second and third order elastic constants and the associated relaxations. Note that the calculation of pressure dependence of elastic constants are presented to emphasize the effect of pressure. For all non-linear elasticity calculations of relative phase stabilities, we use the elastic constants fitted about the stress free configuration of the corresponding phase.

Levenshtein similarity: Consistent prediction of energy ordering of oriented grains from nonlinear elasticity and DFT

Our goal here is to establish that, the ordering of internal energies of grains predicted by nonlinear elasticity is in reasonable agreement with DFT. Our expectation is that at smaller stresses, nonlinear elasticity would be in a very close agreement with DFT predictions and becomes less accurate with increasing stresses.

To quantify this, we need a metric that when given the internal energies predicted by nonlinear elasticity and DFT for a set of grain orientations subjected to a principal stress, should give 1, if they are perfectly in order and 0 if they are completely out of order. To this end we use the concept of Levenshtein distance between strings. (Strings because the ordering of a set of named entities i.e. in our case grains of different orientations, can be represented by strings).

The Levenshtein distance [1] $\mathsf{lev}_{(a,b)}(|a|,|b|)$ is a metric to measure the distance between two strings a and b and represents the smallest number of edit operations to transform one string into the other. The edit operations can be insertions, deletions and substitutions. It is obtained recursively with the formula,

$$\mathsf{lev}_{(a,b)}(i,j) = \begin{cases} \mathsf{max}(i,j) & \text{if } \mathsf{min}(i,j)=0, \\ \min \begin{cases} \mathsf{lev}_{(a,b)}(i-1,j)+1 \\ \mathsf{lev}_{(a,b)}(i,j-1)+1 \\ \mathsf{lev}_{(a,b)}(i-1,j-1)+1_{(a_i \neq b_j)} \end{cases} & \text{otherwise.} \end{cases} \tag{S.1}$$

where i and j are indices to the last character of the sub-strings being compared.

The Leveneshtein similarity ratio is defined by,

$$\frac{|a|+|b|-\mathsf{lev}_{(a,b)}(i,j)}{|a|+|b|} \tag{S.2}$$

where $|a|$ and $|b|$ are the lengths of the strings a and b. The similarity ratio varies between 0 for completely dissimilar to 1 for a perfect match between the strings.

When comparing the ordering of DFT and nonlinear elasticity predicted energy values, we only deal with permutation ordering of the strings and thus no insertions or deletions are present in the edit operations.

In order to compare the ordering of internal energies from elasticity and DFT predictions, we do the following at each point in the stress space:

1. Construct the distribution of internal energies of variously oriented grains (sampling $SO(3)$) of a phase using nonlinear elasticity and pick at least n grain orientations that result in fairly uniformly spaced grain energies, of some "preset" energy spacing $\Delta\Psi$) over the entire spread of the distribution. The choice of the energy spacing is determined by the methods used to compute the grain energies. Grain orientations that result in very close energies cannot be resolved owing to the limits of accuracy of DFT and the inherent errors involved in fitting elastic constants to DFT data.

2. We now have at least n grain orientations and their internal energies from elasticity. For each of these grains at the given stress, compute the internal energies from DFT.

3. Compare the similarity in ordering of elasticity and DFT using Levenshtein similarity ratio.

Supplementary Fig. 10(a) shows the energies of grains of randomly chosen grain orientations from DFT (magenta triangles) and third order elasticity (green circles) for several randomly chosen deviatoric stresses. These results indicate that elasticity provides an excellent ordering of the energies of differently oriented grains. A more quantitative measure of the ordering of a large number of such calculations (sampling the entire deviatoric plane) is through the information-theoretic Levenshtein similarity as discussed above. Supplementary Fig. 10(b) shows the average Levenshtein similarity of energies determined from DFT and elasticity for stress points sampling on the deviatoric plane as a function of deviatoric radius τ. Similarity of $1.0/0$ indicates perfect/no correlation of the orderings of the energies for the two methods for different orientations. These results confirm that nonlinear elasticity satisfactorily predicts the ordering of the energies for different crystal orientations (compared with DFT) for the range of deviatoric stresses of interest here.

Calculation of transformation strain for basal orientation relation

The basal fcc to hcp orientation relationship is given by,

$$\{111\}_{\mathsf{fcc}} \parallel \{0001\}_{\mathsf{hcp}}, \quad \langle 1\bar{1}0\rangle_{\mathsf{fcc}} \parallel \langle 11\bar{2}0\rangle_{\mathsf{hcp}}, \quad \text{and} \quad \langle 11\bar{2}\rangle_{\mathsf{fcc}} \parallel \langle 1\bar{1}00\rangle_{\mathsf{hcp}}. \tag{S.3}$$

The orientational variants are given explicitly below:

Variant	Parallel planes $\boldsymbol{x}_3 \parallel \boldsymbol{y}_3$	Parallel directions $\boldsymbol{x}_1 \parallel \boldsymbol{y}_1$	Parallel directions $\boldsymbol{x}_2 \parallel \boldsymbol{y}_2$
V_1^+	$(111)_{\mathsf{fcc}} \parallel (0001)_{\mathsf{hcp}}$	$[1\bar{1}0]_{\mathsf{fcc}} \parallel [11\bar{2}0]_{\mathsf{hcp}}$	$[11\bar{2}]_{\mathsf{fcc}} \parallel [1\bar{1}00]_{\mathsf{hcp}}$
V_1^-	$(111)_{\mathsf{fcc}} \parallel (0001)_{\mathsf{hcp}}$	$[\bar{1}10]_{\mathsf{fcc}} \parallel [11\bar{2}0]_{\mathsf{hcp}}$	$[\bar{1}\bar{1}2]_{\mathsf{fcc}} \parallel [1\bar{1}00]_{\mathsf{hcp}}$
V_2^+	$(111)_{\mathsf{fcc}} \parallel (0001)_{\mathsf{hcp}}$	$[\bar{1}01]_{\mathsf{fcc}} \parallel [11\bar{2}0]_{\mathsf{hcp}}$	$[1\bar{2}1]_{\mathsf{fcc}} \parallel [1\bar{1}00]_{\mathsf{hcp}}$
V_2^-	$(111)_{\mathsf{fcc}} \parallel (0001)_{\mathsf{hcp}}$	$[10\bar{1}]_{\mathsf{fcc}} \parallel [11\bar{2}0]_{\mathsf{hcp}}$	$[\bar{1}2\bar{1}]_{\mathsf{fcc}} \parallel [1\bar{1}00]_{\mathsf{hcp}}$
V_3^+	$(111)_{\mathsf{fcc}} \parallel (0001)_{\mathsf{hcp}}$	$[01\bar{1}]_{\mathsf{fcc}} \parallel [11\bar{2}0]_{\mathsf{hcp}}$	$[\bar{2}11]_{\mathsf{fcc}} \parallel [1\bar{1}00]_{\mathsf{hcp}}$
V_3^-	$(111)_{\mathsf{fcc}} \parallel (0001)_{\mathsf{hcp}}$	$[0\bar{1}1]_{\mathsf{fcc}} \parallel [11\bar{2}0]_{\mathsf{hcp}}$	$[2\bar{1}\bar{1}]_{\mathsf{fcc}} \parallel [1\bar{1}00]_{\mathsf{hcp}}$
V_4^+	$(1\bar{1}\bar{1})_{\mathsf{fcc}} \parallel (0001)_{\mathsf{hcp}}$	$[\bar{1}\bar{1}0]_{\mathsf{fcc}} \parallel [11\bar{2}0]_{\mathsf{hcp}}$	$[\bar{1}1\bar{2}]_{\mathsf{fcc}} \parallel [1\bar{1}00]_{\mathsf{hcp}}$
V_4^-	$(1\bar{1}\bar{1})_{\mathsf{fcc}} \parallel (0001)_{\mathsf{hcp}}$	$[110]_{\mathsf{fcc}} \parallel [11\bar{2}0]_{\mathsf{hcp}}$	$[1\bar{1}2]_{\mathsf{fcc}} \parallel [1\bar{1}00]_{\mathsf{hcp}}$
V_5^+	$(1\bar{1}\bar{1})_{\mathsf{fcc}} \parallel (0001)_{\mathsf{hcp}}$	$[101]_{\mathsf{fcc}} \parallel [11\bar{2}0]_{\mathsf{hcp}}$	$[\bar{1}\bar{2}1]_{\mathsf{fcc}} \parallel [1\bar{1}00]_{\mathsf{hcp}}$
V_5^-	$(1\bar{1}\bar{1})_{\mathsf{fcc}} \parallel (0001)_{\mathsf{hcp}}$	$[\bar{1}0\bar{1}]_{\mathsf{fcc}} \parallel [11\bar{2}0]_{\mathsf{hcp}}$	$[12\bar{1}]_{\mathsf{fcc}} \parallel [1\bar{1}00]_{\mathsf{hcp}}$
V_6^+	$(1\bar{1}\bar{1})_{\mathsf{fcc}} \parallel (0001)_{\mathsf{hcp}}$	$[01\bar{1}]_{\mathsf{fcc}} \parallel [11\bar{2}0]_{\mathsf{hcp}}$	$[211]_{\mathsf{fcc}} \parallel [1\bar{1}00]_{\mathsf{hcp}}$
V_6^-	$(1\bar{1}\bar{1})_{\mathsf{fcc}} \parallel (0001)_{\mathsf{hcp}}$	$[0\bar{1}1]_{\mathsf{fcc}} \parallel [11\bar{2}0]_{\mathsf{hcp}}$	$[\bar{2}\bar{1}1]_{\mathsf{fcc}} \parallel [1\bar{1}00]_{\mathsf{hcp}}$
V_7^+	$(\bar{1}1\bar{1})_{\mathsf{fcc}} \parallel (0001)_{\mathsf{hcp}}$	$[\bar{1}\bar{1}0]_{\mathsf{fcc}} \parallel [11\bar{2}0]_{\mathsf{hcp}}$	$[\bar{1}12]_{\mathsf{fcc}} \parallel [1\bar{1}00]_{\mathsf{hcp}}$
V_7^-	$(\bar{1}1\bar{1})_{\mathsf{fcc}} \parallel (0001)_{\mathsf{hcp}}$	$[110]_{\mathsf{fcc}} \parallel [11\bar{2}0]_{\mathsf{hcp}}$	$[1\bar{1}\bar{2}]_{\mathsf{fcc}} \parallel [1\bar{1}00]_{\mathsf{hcp}}$
V_8^+	$(\bar{1}1\bar{1})_{\mathsf{fcc}} \parallel (0001)_{\mathsf{hcp}}$	$[\bar{1}01]_{\mathsf{fcc}} \parallel [11\bar{2}0]_{\mathsf{hcp}}$	$[121]_{\mathsf{fcc}} \parallel [1\bar{1}00]_{\mathsf{hcp}}$
V_8^-	$(\bar{1}1\bar{1})_{\mathsf{fcc}} \parallel (0001)_{\mathsf{hcp}}$	$[10\bar{1}]_{\mathsf{fcc}} \parallel [11\bar{2}0]_{\mathsf{hcp}}$	$[\bar{1}\bar{2}\bar{1}]_{\mathsf{fcc}} \parallel [1\bar{1}00]_{\mathsf{hcp}}$
V_9^+	$(\bar{1}1\bar{1})_{\mathsf{fcc}} \parallel (0001)_{\mathsf{hcp}}$	$[011]_{\mathsf{fcc}} \parallel [11\bar{2}0]_{\mathsf{hcp}}$	$[21\bar{1}]_{\mathsf{fcc}} \parallel [1\bar{1}00]_{\mathsf{hcp}}$
V_9^-	$(\bar{1}1\bar{1})_{\mathsf{fcc}} \parallel (0001)_{\mathsf{hcp}}$	$[0\bar{1}\bar{1}]_{\mathsf{fcc}} \parallel [11\bar{2}0]_{\mathsf{hcp}}$	$[\bar{2}11]_{\mathsf{fcc}} \parallel [1\bar{1}00]_{\mathsf{hcp}}$
V_{10}^+	$(\bar{1}\bar{1}1)_{\mathsf{fcc}} \parallel (0001)_{\mathsf{hcp}}$	$[1\bar{1}0]_{\mathsf{fcc}} \parallel [11\bar{2}0]_{\mathsf{hcp}}$	$[112]_{\mathsf{fcc}} \parallel [1\bar{1}00]_{\mathsf{hcp}}$
V_{10}^-	$(\bar{1}\bar{1}1)_{\mathsf{fcc}} \parallel (0001)_{\mathsf{hcp}}$	$[\bar{1}10]_{\mathsf{fcc}} \parallel [11\bar{2}0]_{\mathsf{hcp}}$	$[\bar{1}\bar{1}\bar{2}]_{\mathsf{fcc}} \parallel [1\bar{1}00]_{\mathsf{hcp}}$
V_{11}^+	$(\bar{1}\bar{1}1)_{\mathsf{fcc}} \parallel (0001)_{\mathsf{hcp}}$	$[101]_{\mathsf{fcc}} \parallel [11\bar{2}0]_{\mathsf{hcp}}$	$[\bar{1}21]_{\mathsf{fcc}} \parallel [1\bar{1}00]_{\mathsf{hcp}}$
V_{11}^-	$(\bar{1}\bar{1}1)_{\mathsf{fcc}} \parallel (0001)_{\mathsf{hcp}}$	$[\bar{1}0\bar{1}]_{\mathsf{fcc}} \parallel [11\bar{2}0]_{\mathsf{hcp}}$	$[1\bar{2}\bar{1}]_{\mathsf{fcc}} \parallel [1\bar{1}00]_{\mathsf{hcp}}$
V_{12}^+	$(\bar{1}\bar{1}1)_{\mathsf{fcc}} \parallel (0001)_{\mathsf{hcp}}$	$[011]_{\mathsf{fcc}} \parallel [11\bar{2}0]_{\mathsf{hcp}}$	$[\bar{2}1\bar{1}]_{\mathsf{fcc}} \parallel [1\bar{1}00]_{\mathsf{hcp}}$
V_{12}^-	$(\bar{1}\bar{1}1)_{\mathsf{fcc}} \parallel (0001)_{\mathsf{hcp}}$	$[0\bar{1}\bar{1}]_{\mathsf{fcc}} \parallel [11\bar{2}0]_{\mathsf{hcp}}$	$[2\bar{1}1]_{\mathsf{fcc}} \parallel [1\bar{1}00]_{\mathsf{hcp}}$

These orientation relations are also valid for the reverse phase transformation from hcp to fcc.
From DFT calculations, we determine the equilibrium lattice parameters of fcc and hcp phases of Ni as:

Phase	Lattice parameters (Å)		Volume per atom (Å^3)
fcc	a: 3.51978		10.90165
hcp	a: 2.48595	c: 4.08845	10.94058

From DFT calculations, we determine the equilibrium lattice parameters of hcp and fcc phases of Ti as:

Phase	Lattice parameters (Å)		Volume per atom (Å^3)
hcp	a: 2.93952	c: 4.64588	17.38284
fcc	a: 4.11195		17.38151

Evaluation of lattice correspondence and the stress free transformation deformation gradients $\boldsymbol{F}_t^{\mathsf{fcc} \to \mathsf{hcp}}$ in the case of Ni and $\boldsymbol{F}_t^{\mathsf{hcp} \to \mathsf{fcc}}$ in the case of Ti, are straightforward and are very well documented in the literature, so here we provide one such reference [2] and the lattice parameter values obtained from DFT. To explain briefly (e.g. fcc $\to$ hcp): The stress free transformation deformation gradient can be decomposed into two components; A dilatational strain isotropic in $[111]$ plane associated with an increase in $[111]$ inter-planar spacing, Shear strain due to the shuffling of $\cdots$ABCABCABC$\cdots$ order in FCC to $\cdots$ABABAB$\cdots$ order in HCP on alternate planes. Fix the global orthonormal coordinate system x_1–x_2–x_3 to coincide with the conventional cell of fcc lattice as:

$$x_1 = [100], \quad x_2 = [010], \quad x_3 = [001].$$

The local coordinate system y_1–y_2–y_3 depends on the variant. For the first variant, we use y_1 along $[1\bar{1}0]$ of fcc, y_2 along $[11\bar{2}]$ of fcc and y_3 along $[111]$ of fcc. This corresponds to y_1 along $[11\bar{2}0]$ of hcp, y_2 along $[\bar{1}100]$ of hcp, y_3 along $[0001]$ of hcp. For the isotropic in-plane dilatation of $[111]$ plane, the lattice correspondence is $\frac{1}{\sqrt{2}}a_{\mathsf{fcc}} \to a_{\mathsf{hcp}}$

and in the normal direction $\frac{1}{\sqrt{3}}a_{\text{fcc}} \to \frac{1}{2}c_{\text{hcp}}$. With these lattice correspondences the dilatational component can be evaluated as,

$$\boldsymbol{F}_t^{\text{dil: fcc}\to\text{hcp}} = \begin{bmatrix} \delta_{\text{inplane}} & 0 & 0 \\ 0 & \delta_{\text{inplane}} & 0 \\ 0 & 0 & \delta_{\text{normal}} \end{bmatrix} \tag{S.4}$$

where $\delta_{\text{inplane}} = \sqrt{2}a_{\text{hcp}}/a_{\text{fcc}}$ and $\delta_{\text{normal}} = \frac{\sqrt{3}}{2}c_{\text{hcp}}/a_{\text{fcc}}$. The shear component is given by,

$$\boldsymbol{F}_t^{\text{shear: fcc}\to\text{hcp}} = \begin{bmatrix} 1 & 0 & 0 \\ 0 & 1 & s \\ 0 & 0 & 1 \end{bmatrix} \tag{S.5}$$

where $s = \frac{1}{\sqrt{3}}a_{\text{hcp}}/c_{\text{hcp}}$. The total stress free transformation deformation gradient is given by $\boldsymbol{F}_t^{\text{shear: fcc}\to\text{hcp}}\boldsymbol{F}_t^{\text{dil: fcc}\to\text{hcp}}$ which is then transformed back into the global coordinate system x_1–x_2–x_3. Orientational variants and the reverse transformation $\boldsymbol{F}_t^{\text{hcp}\to\text{fcc}}$ can be handled in a similar fashion.

Calculation of transformation strain for Burgers orientation relation

The bcc to hcp Burgers orientation relationship is given by,

$$\{1\bar{1}0\}_{\text{bcc}} \parallel \{0001\}_{\text{hcp}}, \quad \langle 111\rangle_{\text{bcc}} \parallel \langle 11\bar{2}0\rangle_{\text{hcp}}, \quad \text{and} \quad \langle\bar{1}\bar{1}2\rangle_{\text{bcc}} \parallel \langle\bar{1}100\rangle_{\text{hcp}}. \tag{S.6}$$

The orientational variants are given below:

Variant	Parallel planes $\boldsymbol{x}_3 \parallel \boldsymbol{y}_3$	Parallel directions $\boldsymbol{x}_1 \parallel \boldsymbol{y}_1$	Parallel directions $\boldsymbol{x}_2 \parallel \boldsymbol{y}_2$
V_1	$(110)_{\text{bcc}} \parallel (0001)_{\text{hcp}}$	$[\bar{1}1\bar{1}]_{\text{bcc}} \parallel [11\bar{2}0]_{\text{hcp}}$	$[\bar{1}12]_{\text{bcc}} \parallel [\bar{1}100]_{\text{hcp}}$
V_2		$[1\bar{1}\bar{1}]_{\text{bcc}} \parallel [11\bar{2}0]_{\text{hcp}}$	$[\bar{1}1\bar{2}]_{\text{bcc}} \parallel [\bar{1}100]_{\text{hcp}}$
V_3	$(1\bar{1}0)_{\text{bcc}} \parallel (0001)_{\text{hcp}}$	$[11\bar{1}]_{\text{bcc}} \parallel [11\bar{2}0]_{\text{hcp}}$	$[112]_{\text{bcc}} \parallel [\bar{1}100]_{\text{hcp}}$
V_4		$[111]_{\text{bcc}} \parallel [11\bar{2}0]_{\text{hcp}}$	$[\bar{1}\bar{1}2]_{\text{bcc}} \parallel [\bar{1}100]_{\text{hcp}}$
V_5	$(011)_{\text{bcc}} \parallel (0001)_{\text{hcp}}$	$[11\bar{1}]_{\text{bcc}} \parallel [11\bar{2}0]_{\text{hcp}}$	$[\bar{2}1\bar{1}]_{\text{bcc}} \parallel [\bar{1}100]_{\text{hcp}}$
V_6		$[\bar{1}1\bar{1}]_{\text{bcc}} \parallel [11\bar{2}0]_{\text{hcp}}$	$[21\bar{1}]_{\text{bcc}} \parallel [\bar{1}100]_{\text{hcp}}$
V_7	$(01\bar{1})_{\text{bcc}} \parallel (0001)_{\text{hcp}}$	$[\bar{1}11]_{\text{bcc}} \parallel [11\bar{2}0]_{\text{hcp}}$	$[211]_{\text{bcc}} \parallel [\bar{1}100]_{\text{hcp}}$
V_8		$[111]_{\text{bcc}} \parallel [11\bar{2}0]_{\text{hcp}}$	$[2\bar{1}\bar{1}]_{\text{bcc}} \parallel [\bar{1}100]_{\text{hcp}}$
V_9	$(101)_{\text{bcc}} \parallel (0001)_{\text{hcp}}$	$[\bar{1}11]_{\text{bcc}} \parallel [11\bar{2}0]_{\text{hcp}}$	$[\bar{1}\bar{2}1]_{\text{bcc}} \parallel [\bar{1}100]_{\text{hcp}}$
V_{10}		$[11\bar{1}]_{\text{bcc}} \parallel [11\bar{2}0]_{\text{hcp}}$	$[\bar{1}21]_{\text{bcc}} \parallel [\bar{1}100]_{\text{hcp}}$
V_{11}	$(\bar{1}01)_{\text{bcc}} \parallel (0001)_{\text{hcp}}$	$[1\bar{1}1]_{\text{bcc}} \parallel [11\bar{2}0]_{\text{hcp}}$	$[121]_{\text{bcc}} \parallel [\bar{1}100]_{\text{hcp}}$
V_{12}		$[111]_{\text{bcc}} \parallel [11\bar{2}0]_{\text{hcp}}$	$[\bar{1}2\bar{1}]_{\text{bcc}} \parallel [\bar{1}100]_{\text{hcp}}$

From DFT calculations, we determine the equilibrium lattice parameters of bcc and hcp phases of Fe as:

Phase	Lattice parameters (Å)		Volume per atom (Å^3)
bcc	a: 2.83103		11.34471
hcp	a: 2.45621	c: 3.880361	10.13688

We follow the reference DeGraef [3] to derive the stress free transformation deformation gradient $\boldsymbol{F}_t$ for the phase transformation from bcc to hcp phase in Fe. We briefly outline the procedure here: Fix the global orthonormal coordinate system x_1–x_2–x_3 to coincide with the conventional cell of bcc lattice as:

$$x_1 = [100], \quad x_2 = [010], \quad x_3 = [001].$$

For the first variant, the local orthonormal coordinate basis y_1–y_2–y_3 is taken as follows: $[1\bar{1}\bar{2}]_{\text{bcc}} \parallel [1\bar{1}00]_{\text{hcp}}$ as y_1 axis, $[\bar{1}1\bar{1}]_{\text{bcc}} \parallel [11\bar{2}0]_{\text{hcp}}$ as y_2 axis $(110)_{\text{bcc}} \parallel (0001)_{\text{hcp}}$ as y_3 axis.

The lattice correspondence is given as follows: Along y_2: $\frac{\sqrt{3}}{2}a_{\text{bcc}} \to a_{\text{hcp}}$, along y_3 : $\sqrt{2}a_{\text{bcc}} \to c_{\text{hcp}}$. The third lattice correspondence is not orthogonal to the above two. $[1\bar{1}\bar{1}]_{\text{bcc}}$ maps to $[1\bar{2}10]_{\text{hcp}}$ (the lattice correspondence lengths are same as that of along y_2). Note that $[1\bar{1}\bar{1}]_{\text{bcc}}$ makes $109.47° - 90° = 10.47°$ with y_1 or equivalently $[1\bar{2}10]_{\text{hcp}}$ makes $30°$ with y_1. With this information and following section 1.9.5 in DeGraef[3] we can evaluate $\boldsymbol{F}_t^{\text{bcc}\to\text{hcp}}$ in the x_1–x_2–x_3 basis as:

$$\boldsymbol{F}_t^{\text{bcc}\to\text{hcp}} = \begin{bmatrix} 0.92023 & 0 & 0 \\ -0.17709 & 1.00182 & 0 \\ 0 & 0 & 0.96919 \end{bmatrix} \tag{S.7}$$

A necessary (but not sufficient) condition to cross check is that $J = \det \boldsymbol{F}_t^{\text{bcc}\to\text{hcp}} = 0.8935 = V_{\text{hcp}}^{\text{stress-free}}/V_{\text{bcc}}^{\text{stress-free}} = 10.13688/11.34471$ should be satisfied. The deformation gradients of other variants can be derived accordingly through basis transformation.

Identification of phase transformation (PT) onset boundaries

Discretization of stress space

We sample the stress space at regular intervals of pressure with a step size of (i) 1 GPa in the range of -5 GPa to 5 GPa for both Ni and Ti and (ii) 0.5 GPa in the range of 0 to 10 GPa for Fe, with some additional points around the isenthalpic coexistence pressure of 9.58 GPa as evident from Fig. 3 in main text between 8.5 GPa and 9.58 GPa. Deviatoric planes are constructed at these discrete pressure levels. Since the shear stresses needed to induce PTs are not known a priori; we consider large circular regions with a radius of 8 GPa normal to the hydrostatic axis i.e. the deviatoric plane. This region is discretized in 20 MPa steps in the radial direction and in $4°$ steps in the angular direction in the minimal circular sector of $0°$ to $60°$ (recall the three fold and mirror symmetries of stress space). A coarse grid along the p-axis is sufficient as we are only interested in the dependence of minimum deviatoric stress needed to induce PT on the pressure. On the other hand, each deviatoric plane is discretized finely, to accurately calculate the onset of PT boundaries from nonlinear elasticity. Once the PT onset boundaries and the corresponding parent and daughter grain orientations are determined using nonlinear elasticity for all deviatoric planes, we refine these predictions using DFT as explained in the next section.

Stress space exploration

First, each point in the stress space corresponds to the principal stress triplet $(\sigma_1, \sigma_2, \sigma_3)$ of the Cauchy stress. Assume a global Cartesian reference frame x_i° with basis $\boldsymbol{e}_i^\circ$ attached to this stress space. Crystals/grains of various orientations can be described with respect to this global reference frame . Each oriented crystal/grain has a local lattice cartesian frame x_i with basis $\boldsymbol{e}_i$ attached to it. Thus we create a transformation matrix $T_{ij} = \cos(x_i, x_j^\circ)$ such that $\boldsymbol{e}_i = T_{ij}\boldsymbol{e}_j^\circ$; the stress state $\boldsymbol{\sigma}$ in the grain frame is given by $\sigma_{ij} = T_{ip}T_{jq}\sigma_{pq}^\circ$ where $\boldsymbol{\sigma}^\circ =$ diagonal matrix$(\sigma_1, \sigma_2, \sigma_3)$. This establishes the stress state in the grain frame and is the usual tensor transformation under a change of Cartesian basis.

The second part is the exploration of the stress space. In the initial stage of our calculations (based on nonlinear elasticity), the calculation is straight forward in the sense that at each point in the stress space, we consider **all** possible grain orientations of parent and its transformation daughter variant and compute the Gibbs free energies. Thus our exploration of crystal orientations for each stress is complete; no parts of the orientation space are omitted nor are any points in the stress space omitted.

The next step in our work is the refining of the transformation onset boundary using DFT. Once a particular point on the transformation boundary in stress space $(r, \theta, p) \equiv (\sigma_1, \sigma_2, \sigma_3)$ and associated parent and daughter grain orientations (identified in our earlier elasticity calculation), we perform a 1D line search (for the particular p and θ). That is, on the deviatoric plane associated with pressure p, we march radially at a particular θ, constructing Cauchy stresses along the radial path and performing DFT based transformation prediction check at each point along the radial line. Please note, that such a radial exploration is performed for each $\theta \in [0, 60°]$ on the deviatoric plane and for all deviatoric planes in a range of pressures considered.

Radial continuation scheme

On each deviatoric plane, we have phase transformation onset boundaries determined using nonlinear elasticity. Each point on these boundaries may correspond to multiple pairs of grains (parent-daughter variants). At each θ, if a solution (PT boundary from nonlinear elasticity) exists, we choose the highest enthalpy parent grain orientation $\boldsymbol{g}_{\text{el}}^{\alpha}$ among all solutions and "its" daughter variant $\boldsymbol{g}_{\text{el}}^{\beta}$ with lowest enthalpy. The corresponding radius be r_{el}. Now our goal is to determine r_{DFT}?

This is achieved through the process of radial continuation, schematically depicted in Supplementary Fig. 11. Let us choose a point E on the elastic PT onset boundary. The coordinates of this point in the stress space are given by $\widetilde{\sigma}_{\text{el}} = (r_{\text{el}}, \theta, p)$. The position vector of this point in stress space can be decomposed into orthogonal components, $\overrightarrow{\text{ON}}$ along the hydrostatic axis and $\overrightarrow{\text{NE}}$ perpendicular to the hydrostatic axis and lying in the corresponding deviatoric plane, where O is the origin of the stress space.

Assume grain orientations $\mathbf{g}_{\text{DFT}}^{\alpha} = \mathbf{g}_{\text{el}}^{\alpha}$ and $\mathbf{g}_{\text{DFT}}^{\beta} = \mathbf{g}_{\text{el}}^{\beta}$. On this deviatoric plane (p held constant) and along the direction θ represented by the unit vector $\hat{\boldsymbol{d}}$, we increment r in steps, marching outwards from the point N towards E as below:

$$\sigma_r = \overrightarrow{\text{ON}} + r\hat{\boldsymbol{d}}, \quad \hat{\boldsymbol{d}} = \frac{\overrightarrow{\text{NE}}}{\|\overrightarrow{\text{NE}}\|}, \quad r \geq 0. \tag{S.8}$$

At each point $\sigma_r = (r, \theta, p)$ along the path, calculate $h^{\alpha}(\boldsymbol{\sigma}(\sigma(r,\theta,p), \mathbf{g}_{\text{DFT}}^{\alpha}))$ and $h^{\beta}(\boldsymbol{\sigma}(\sigma(r,\theta,p), \mathbf{g}_{\text{DFT}}^{\beta}), \boldsymbol{F}_t)$ of the oriented α and β grains using DFT, and check whether the condition $h^{\beta}(r) \leq h^{\alpha}(r)$ is satisfied. If the condition is satisfied, we have found the DFT PT onset radius, r_{DFT} at the chosen θ and p i.e $\widetilde{\sigma}_{\text{DFT}} = (r_{\text{DFT}}, \theta, p)$. The locus of all such points corresponds to the DFT-based PT onset boundary on each deviatoric plane. In the case where (i) α and/or β grains lose stability before they intersect or (ii) their enthalpy curves do not intersect or (iii) there is no nonlinear elastic solution to begin with; there is no PT in that direction, this is schematically indicated as "Fail" (i.e., the search process can find no solution) in the Supplementary Fig. 11. When marching along the radial direction from a radius r_i to the next step r_{i+1}, we use the converged atomic configuration at r_i (under a stress $\widetilde{\sigma}_{r_i}$) as a starting configuration at r_{i+1} to be subjected to an incremented stress $\widetilde{\sigma}_{r_{i+1}}$ to speed up the convergence process. This is akin to the procedure of numerical continuation algorithms where a control parameter that parameterizes the arc-length along the path is incremented and hence the name radial continuation.

Application of Cauchy stress in elasticity framework

A computational material model can be seen as a black-box that mimics the constitutive behavior, i.e. in simplistic terms, given a strain what is the stress or vice versa. In case of molecular simulation methods this constitutive relation is reproduced by the inter-atomic force fields, in case of DFT it is based on pseudo potentials and solving the Kohn–Sham equations. Another simple and widely used method to represent the material behavior (we limit to zero kelvin here) is through the construction of Taylor series expansion of the internal energy as a function of strain. The coefficients of the expansion are fitted to reproduce material behavior from a more accurate theory which in our case is DFT. Once we have these coefficients we can predict the material behavior of course in some range of stresses and strains and with varying accuracy. Our goal in this section is to explain a simple algorithm to predict the material response (strain) for a prescribed stress in the deformed configuration (Cauchy stress).

The internal energy density of a strained configuration can be expressed as an expansion about the unstrained-stress free reference configuration in terms of the Green-Lagrange strain tensor $\boldsymbol{E}$ as,

$$\rho_0 U(\boldsymbol{E}) = \rho_0 U_0(\boldsymbol{0}) + \frac{1}{2!}\boldsymbol{E} : \mathbb{C}^{\mathrm{II}} : \boldsymbol{E} + \frac{1}{3!}\left(\boldsymbol{E} : \mathbb{C}^{\mathrm{III}} : \boldsymbol{E}\right) : \boldsymbol{E} + \frac{1}{4!}\boldsymbol{E} : \left(\boldsymbol{E} : \mathbb{C}^{\mathrm{IV}} : \boldsymbol{E}\right) : \boldsymbol{E} + \cdots \tag{S.9}$$

where $\mathbb{C}^{\mathrm{II}}, \mathbb{C}^{\mathrm{III}}$ and $\mathbb{C}^{\mathrm{IV}}$ are the second, third and fourth order isentropic elastic tensors. In the current work, we perform simulations only at <u>zero kelvin</u>, so we neglect the distinction between adiabatic and isothermal elastic constants. Also we limit the expansion of strain energy to <u>third order</u>.

The second Piola-Kirchof (PK) stress $\boldsymbol{S}$ in the component form is given by,

$$S_{ij} = \rho_0 \frac{\partial U}{\partial E_{ij}} = \mathbb{C}_{ijkl} E_{kl} + \frac{1}{2}\mathbb{C}_{ijklmn} E_{kl} E_{mn} + \cdots \tag{S.10}$$

In molecular statics/DFT simulations, application of strain $\boldsymbol{E}$ is not done directly but rather through mapping the simulation cell to the deformed configuration through the deformation gradient tensor $\boldsymbol{F}$. This is done through inverting the relation,

$$\boldsymbol{E} = \frac{1}{2}(\boldsymbol{C} - \boldsymbol{I}) \tag{S.11}$$

where $\boldsymbol{C} = \boldsymbol{F}^T \boldsymbol{F}$. Recall that the idea behind using $\boldsymbol{C}$ in defining $\boldsymbol{E}$ through Eq. S.11, the information about the rigid rotation present in $\boldsymbol{F}$ is destroyed. Thus the inverse solution is non-unique with respect to arbitrary rigid rotations and of course rigid translations. But since the strain energy is frame independent, rigid rotations do not matter. We do this inversion uniquely (rather consistently) by constructing the symmetric form $\widetilde{\boldsymbol{F}}$ of $\boldsymbol{F}$ as:

$$\boldsymbol{C} = 2\boldsymbol{E} + \boldsymbol{I} = \boldsymbol{Q}\boldsymbol{\lambda}\boldsymbol{Q}^T \quad \text{since } \boldsymbol{C} \text{ is diagonalizable} \tag{S.12}$$

$$\widetilde{\boldsymbol{F}} = \sqrt{\boldsymbol{C}} = \boldsymbol{Q}\sqrt{\boldsymbol{\Lambda}}\boldsymbol{Q}^T \tag{S.13}$$

where is $\boldsymbol{Q}$ is the matrix of eigenvectors of $\boldsymbol{C}$ and is orthogonal. $\boldsymbol{\Lambda}$ is a diagonal matrix with diagonal entries as the eigenvalues of $\boldsymbol{C}$. For brevity of further discussion, we represent the whole process though a shorthand notation,

$$\widetilde{\boldsymbol{F}} = \widetilde{\mathcal{F}}(\boldsymbol{E}) \tag{S.14}$$

As we can see that the elastic tensors are relate the Lagrangian strain $\boldsymbol{E}$ to the second PK stress $\boldsymbol{S}$, both defined in the reference configuration. But in this work, we are interested in knowing the deformation and internal strain energy when the stresses are specified in the deformed configuration, usually specified in terms of the symmetric Cauchy stress $\boldsymbol{\sigma}$. Here we explain a simple scheme to achieve this under the framework of elastic tensors. The idea is to iteratively correct the applied strain $\boldsymbol{E}$ (usually $\boldsymbol{F}$ is directly used in simulations to control the deformation of the simulation cell) until the stress in the deformed configuration $\boldsymbol{\sigma}$ converges to the target value $\boldsymbol{\sigma}^{\text{target}}$.

For brevity of notation, let us define two abstract functions; the stiffness function $\mathcal{C}$ and its inverse, the compliance function $\mathcal{S}$. As the names suggest, the stiffness function, when given a strain $\boldsymbol{E}$ returns the second PK stress $\boldsymbol{S}$ (Eq. S.10).

$$\boldsymbol{S} = \mathcal{C}(\boldsymbol{E}) \tag{S.15}$$

The compliance function, does the reverse, i.e. given the second PK stress $\boldsymbol{S}$ returns $\boldsymbol{E}$,

$$\boldsymbol{E} = \mathcal{S}(\boldsymbol{S}) \tag{S.16}$$

In general one can either construct the compliance tensors from elastic (stiffness) tensors [4, 5] or use a numerical predictor-corrector based algorithm using the stiffness function $\mathcal{C}$ to compute the strain $\boldsymbol{E}$ for a given stress $\boldsymbol{S}$.

Finally, our goal is to impose a Cauchy stress on the system where the constitutive response is known in terms of the elastic tensors. We describe an algorithm to converge to the target Cauchy stress with in the tensor tolerance $\boldsymbol{\sigma}^{\text{tol}}$.

Algorithm 1 An algorithm to apply target Cauchy stress

procedure $\textsc{Apply\_Cauchy}(\boldsymbol{\sigma}^{\text{target}}, \boldsymbol{\sigma}^{\text{tol}}; \mathcal{S}, \widetilde{\mathcal{F}})$
  $\boldsymbol{S} \leftarrow \boldsymbol{\sigma}^{\text{target}}$ ▷ This is equivalent to assuming $\boldsymbol{F} = \boldsymbol{I}$
  do
    $\boldsymbol{E} \leftarrow \mathcal{S}(\boldsymbol{S})$
    $\widetilde{\boldsymbol{F}} \leftarrow \widetilde{\mathcal{F}}(\boldsymbol{E})$
    $\boldsymbol{\sigma} \leftarrow \frac{1}{\det \widetilde{\boldsymbol{F}}} \widetilde{\boldsymbol{F}} \boldsymbol{S} \widetilde{\boldsymbol{F}}^T$
    $d\boldsymbol{\sigma} \leftarrow \boldsymbol{\sigma}^{\text{target}} - \boldsymbol{\sigma}$
    $\mathrm{d}\boldsymbol{S} \leftarrow (\det \widetilde{\boldsymbol{F}}) \widetilde{\boldsymbol{F}}^{-1} d\boldsymbol{\sigma} \widetilde{\boldsymbol{F}}^{-T}$
    $\boldsymbol{S} \leftarrow \boldsymbol{S} + d\boldsymbol{S}$
  while $|d\boldsymbol{\sigma}| < \boldsymbol{\sigma}^{\text{tol}}$
  return $\boldsymbol{E}^{\text{target}} \leftarrow \boldsymbol{E}$
end procedure

The strain $\boldsymbol{E}^{\text{target}}$ is the required strain to obtain target Cauchy stress $\boldsymbol{\sigma}^{\text{target}}$. We can then evaluate the internal energy density U at a prescribed $\boldsymbol{\sigma}$ using the elastic tensors as specified in Eq. S.9 in terms of $\boldsymbol{E}$. In the current work we set a strict convergence tolerance of 1Pa on each component of stress in $\boldsymbol{\sigma}^{\text{tol}}$.

Supplementary Notes

A general remark on the enthalpy equation

The enthalpy of a system under hydrostatic stress (pressure p) is given by the well known expression,

$$H = \Psi + pV \tag{S.17}$$

where Ψ is the internal energy of the system, p is the pressure and V is the current volume.

In case of non hydrostatic stress, the expression for enthalpy of a system in the small strain case is usually expressed as,

$$H = \Psi - V_0 \boldsymbol{\sigma} : \boldsymbol{\varepsilon} \tag{S.18}$$

where $\boldsymbol{\sigma}$ and ε are the stress and strain tensors and V_0 is the reference volume.

Note that the above definition does not reduce to the case of enthalpy under hydrostatic stress when the stress is hydrostatic. Several works have discussed this aspect and proposed various ways of computing enthalpy for non-hydrostatic cases [6, 7, 8].

In case of a system undergoing large deformations, the enthalpy density is given by,

$$h(\boldsymbol{P}, \boldsymbol{F}_t) = \psi(\boldsymbol{E}_e) - \boldsymbol{P} : \boldsymbol{F} \tag{S.19}$$

The above relation does not exactly reduce to the small strain expression when the stresses/strains are small. Levitas [9] proposed an alternative enthalpy (Gibbs potential),

$$h(\boldsymbol{P}, \boldsymbol{F}_t) = \psi(\boldsymbol{E}_e) - \boldsymbol{P} : (\boldsymbol{F} - \boldsymbol{I}) \tag{S.20}$$

which in the small strain limit reduces to the corresponding expression. In the current work we adopt this form of enthalpy. Note that although we adopt this formulation, the method proposed to determine phase transition remains the same and any suitable expression for enthalpy could be used.

Supplementary References

[1] Vladimir I Levenshtein. Binary codes capable of correcting deletions, insertions, and reversals. In *Soviet physics doklady*, volume 10, pages 707–710, 1966.

[2] Tae Wook Heo, Kimberly B Colas, Arthur T Motta, and Long-Qing Chen. A phase-field model for hydride formation in polycrystalline metals: Application to δ-hydride in zirconium alloys. *Acta Materialia*, 181:262–277, 2019.

[3] Marc De Graef. *Introduction to conventional transmission electron microscopy*. Cambridge university press, 2003.

[4] Vlado A Lubarda. New estimates of the third-order elastic constants for isotropic aggregates of cubic crystals. *Journal of the Mechanics and Physics of Solids*, 45(4):471–490, 1997.

[5] Christopher M Kube and Joseph A Turner. Estimates of nonlinear elastic constants and acoustic nonlinearity parameters for textured polycrystals. *Journal of Elasticity*, 122(2):157–177, 2016.

[6] Ching Cheng, Wung-Hong Huang, and HJ Li. Thermodynamics of uniaxial phase transition: Ab initio study of the diamond-to-β-tin transition in si and ge. *Physical Review B*, 63(15):153202, 2001.

[7] Katalin Gaál-Nagy and Dieter Strauch. Transition pressures and enthalpy barriers for the cubic diamond$\rightarrow$ β-tin transition in si and ge under nonhydrostatic conditions. *Physical Review B*, 73(13):134101, 2006.

[8] Ambarish J Kulkarni, Min Zhou, Kanoknan Sarasamak, and Sukit Limpijumnong. Novel phase transformation in zno nanowires under tensile loading. *Physical Review Letters*, 97(10):105502, 2006.

[9] Valery I Levitas. Phase-field theory for martensitic phase transformations at large strains. *International Journal of Plasticity*, 49:85–118, 2013.
